# Tailor-made alkaliphilic and thermostable fungal laccases for industrial wood processing

**DOI:** 10.1186/s13068-022-02247-2

**Published:** 2022-12-29

**Authors:** David Rodríguez-Escribano, Rocío Pliego-Magán, Felipe de Salas, Pablo Aza, Patrizia Gentili, Petri Ihalainen, Thomas Levée, Valérie Meyer, Michel Petit-Conil, Sandra Tapin-Lingua, Michael Lecourt, Susana Camarero

**Affiliations:** 1 Centro de Investigaciones Biológicas Margarita Salas, CSIC. Ramiro de Maeztu 9, 28040 Madrid, Spain; 2 Sapienza Università Di Roma, Piazzale Aldo Moro, 5, 00185 Rome, RM Italy; 3 MetGen Oy, Rakentajantie 26, 20780 Kaarina, Finland; 4 Centre Technique du Papier (CTP), Domaine Universitaire, 38044 Grenoble Cedex 9, France; 5 FCBA Institut Technologique, 341 Rue de La Papeterie, 38610 Gières, France

**Keywords:** Laccase, Enzyme directed evolution, Extremophilic properties, Lignin, Kraft pulping, Fibreboard, Biorefinery

## Abstract

**Background:**

During the kraft process to obtain cellulosic pulp from wood, most of the lignin is removed by high-temperature alkaline cooking, released in the black liquors and usually incinerated for energy. However, kraft lignins are a valuable source of phenolic compounds that can be valorized in new bio-based products. The aim of this work is to develop laccases capable of working under the extreme conditions of high temperature and pH, typical of the industrial conversion of wood into kraft pulp and fibreboard, in order to provide extremophilic biocatalysts for depolymerising kraft lignin, and enzyme-assisted technologies for kraft pulp and fibreboard production.

**Results:**

Through systematic enzyme engineering, combining enzyme-directed evolution and rational design, we changed the optimal pH of the laccase for oxidation of lignin phenols from acidic to basic, enhanced the catalytic activity at alkaline pH and increased the thermal tolerance of the enzyme by accumulating up to eight mutations in the protein sequence. The extremophilic laccase variants show maximum activity at 70 °C and oxidize kraft lignin at pH 10. Their integration into industrial-type processes saves energy and chemicals. As a pre-bleaching stage, the enzymes promote kraft pulp bleachability and significantly reduce the need for chlorine dioxide compared to the industrial sequence. Their application in wood chips during fibreboard production, facilitates the defibering stage, with less energy required.

**Conclusions:**

A set of new alkaliphilic and thermophilic fungal laccases has been developed to operate under the extreme conditions of high temperature and pH typical of industrial wood conversion processes. For the first time basidiomycete laccases of high-redox potential show activity on lignin-derived phenols and polymeric lignin at pH 10. Considering the extreme conditions of current industrial processes for kraft pulp and fibreboard production, the new tailor-made laccases constitute a step forward towards turning kraft pulp mills into biorefineries. Their use as biocatalysts in the wood conversion sector is expected to support the development of more environmentally sound and efficient processes, and more sustainable products.

**Supplementary Information:**

The online version contains supplementary material available at 10.1186/s13068-022-02247-2.

## Background

Lignin is the second most abundant polymer in nature as a part of lignocellulose with a heterogeneous aromatic nature. The structure and composition of lignin greatly vary among plant species or tissues, depending on the occurrence and bonding between the three main types of phenylpropanoid units originated from the oxidative coupling of p-coumaryl, coniferyl and sinapyl alcohols [1, 2]. Lignin matrix covers the cellulose microfibrils in the secondary wall of plant cells, acting as a protective barrier against microbial attack and providing structural integrity to the plant. Therefore, for the industrial utilization of cellulose, the lignin polymer has to be removed under harsh extraction conditions due to the recalcitrance of the polymeric structure. In the pulp and paper industry, wood chips are cooked at elevated temperature with sodium sulphide and caustic soda (kraft process) or sulphite salts and sulphur dioxide (sulphite process) to remove lignin and produce chemical pulp. The leftover lignins are solubilized in the black liquors and generally burnt to generate energy for the mill. Nevertheless, lignin is a valuable renewable raw material for the chemical industry, due to its hydrophobic, thermal and binding properties and its chemical aromatic composition.

In the last decade, intensive research has been carried out to transform industrial lignins into added-value products, in line with the global demand for bio-based products to reduce dependence on fossil resources [3]. However, standardization of conversion methods for the valorization of industrial lignins into materials and chemicals is hampered by the heterogeneous chemical structure and polydispersity of these lignins, which are highly dependent on the plant source and the lignin extraction process [4]. Kraft lignins have been particularly unexploited although the kraft process accounts for over 85% of the chemical pulp produced worldwide. Recent developments in the supply of kraft lignins through LignoBoost^®^ [5] or LignoForce™ [6] processes are expected to increase their market share.

The most important emerging applications of industrial lignins involve their depolymerization to generate added-value small molecules as a sustainable alternative to petroleum-derived chemicals [7]. Thermal and chemical depolymerization methods allow attaining several lignin fractions separated according to their molar mass [8]. Besides, biotechnology contributes to the exploitation of this valuable feedstock by providing microbial and enzymatic tools to depolymerize lignin into small oligomers and chemicals.

Among biological approaches, white-rot basidiomycetes efficiently degrade lignin from wood in an oxidative process based on the action of extracellular ligninolytic oxidoreductases in association with auxiliary enzymes and cofactors [9]. In the current lignocellulose biorefinery concept, the ligninolytic system secreted by these fungi, which includes ligninolytic peroxidases, laccases, GMC oxidases and unspecific peroxygenases, constitute powerful biotechnological tools for the integral conversion of plant biomass [10]. Due to the lower efficiency of bacteria to act on high Mw lignins, few studies have reported lignin decomposition by bacteria as compared with those involving ligninolytic fungi. However, recent advances in lignin bioconversion include coupling of chemo-catalytic depolymerization of lignin to low molecular weight species with bacterial catabolism pathways to redirect intracellular aromatic intermediates to unique bioproducts [11, 12]. Biological funnelling of aromatic compounds from lignin breakdown by extracellular microbial enzymes into added-value bioproducts via bacterial metabolism is also possible [13, 14]. Still, conversion of lignin into added-value products is a considerable technical challenge due to the lack of robust and efficient biotechnological tools for breaking down this heterogeneous and recalcitrant polymer.

Among ligninolytic enzymes, laccases present inherent characteristics that may be key to address these challenges, although their cost-efficient industrial production has to be solved [15]. Unlike ligninolytic peroxidases, which require a meticulous dosage of hydrogen peroxide, laccases only need oxygen from the air as sole catalytic requirement and produce water as a by-product. Because of this and their versatility to oxidize lignin [16] and a wide spectrum of phenols, aryl amines and N-heterocycles, they have been the most applied ligninolytic oxidoreductases in different sectors [17–20].

Laccases are multicopper oxidases with four copper ions and ten His and one Cys residues coordinating the coppers that participate in the catalysis. Copper T1 catalyses the oxidation of the reducing substrate. The electrons are sequentially transferred through the copper ligands to the other three copper ions (one T2/two T3) arranged in a trinuclear cluster (TNC) where oxygen is reduced [21]. In low redox potential laccases (LRPLs) found in bacteria and plants, a Met residue acts as the fourth axial ligand of copper T1, whose coordination shows a distorted tetrahedral conformation, whereas in medium redox potential laccases (MRPLs) and high redox potential laccases (HRPLs) secreted by lignin-degrading fungi, this position is occupied by a non-coordinating Phe or Leu residue, making a trigonal planar conformation of the T1 site [22].

Laccases are particularly suitable to act on kraft lignins extracted from the black liquors of kraft pulping due to the strong phenolic nature of these technical lignins. However, the alkaline conditions at which kraft lignins are soluble (pH ≥ 10) hinder their modification by fungal laccases, which have naturally evolved to oxidize lignin in acidic conditions and mild temperatures [23]. Laccases can be also useful to aid pulp bleaching and delignification, or to promote defibering of wood chips or fibre bonding in fibreboard production. These wood conversion applications also require high temperatures and neutral-to-alkaline conditions [24].

Directed evolution is a powerful protein engineering strategy based on iterations of random mutagenesis and selection under desired selective pressure to adjust the intrinsic properties of native enzymes to the more demanding industrial operation conditions or to confer new properties to the enzyme [25]. In previous enzyme-directed evolution campaigns, we have obtained recombinant fungal laccases secreted by *Saccharomyces cerevisiae* designed towards different targets [26, 27] and followed different engineering strategies to improve enzyme production yields by the yeast [28, 29]. Nonetheless, up-scale production in industrial hosts such as *Komagataella pastoris* or *Aspergillus oryzae* is commonly pursued to appraise the biotechnological potential of the engineered enzymes [30, 31].

In this work, using enzyme-directed evolution combined with rational design, we developed fungal laccases produced in yeast that are able to operate at alkaline pH and high temperature, the conditions typical of kraft process and fibreboard manufacture. The contribution of the acquired mutations to the alkaliphilicity and thermal resistance of the new variants is discussed. Finally, the *extremozymes* are tested as biocatalysts for kraft lignin modification, kraft pulp bleaching and fibreboard production.

## Results

### First laccase-directed evolution campaign towards alkaliphilicity: 7A12 lineage

**Fig. 1:**
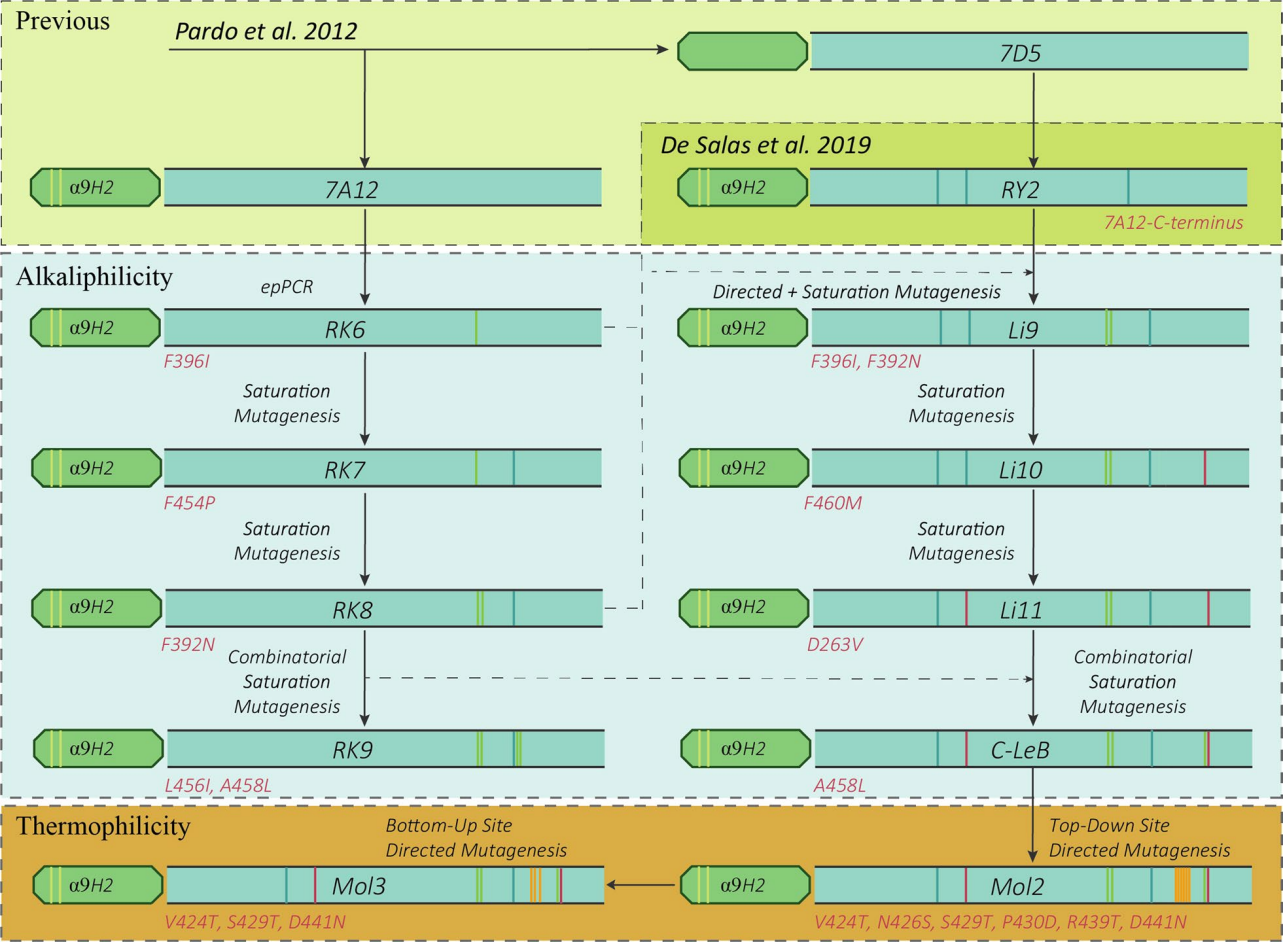
General scheme of laccase engineering towards extremophilicity. Shown are the two evolutionary trajectories from starting 7A12 (left) or RY2 (right) laccases and the selected variants of each lineage. Mutations selected in each evolution round are depicted as vertical lines in green light (for 7A12 lineage) and in red (for RY2 lineage) for alkaliphilicity engineering, and in orange for thermophilicity. Mutations selected during the evolution of 7D5 laccase to obtain RY2 variant are also shown, in the laccase sequence (in blue) or in the evolved α_9H2_ leader (yellow), which is used here as signal peptide to facilitate the secretion of all laccase variants

Laccase 7A12 engineered in *S. cerevisiae* [32] from PM1 basidiomycete laccase (PM1L) [33] was selected as the starting point of the directed evolution campaign towards alkaliphilicity (Fig. 1). We used 2,6-dimethoxyphenol (DMP) and guaiacol to explore the activity on lignin-derived phenols of the mutant libraries expressed in *S. cerevisiae*, and selected the best enzyme variants by their improved activities, first at pH 6, and then at pH 8 and 9. ABTS oxidation at pH 3 was used as a reference of the initial acidic activity of laccase.

**Fig. 2:**
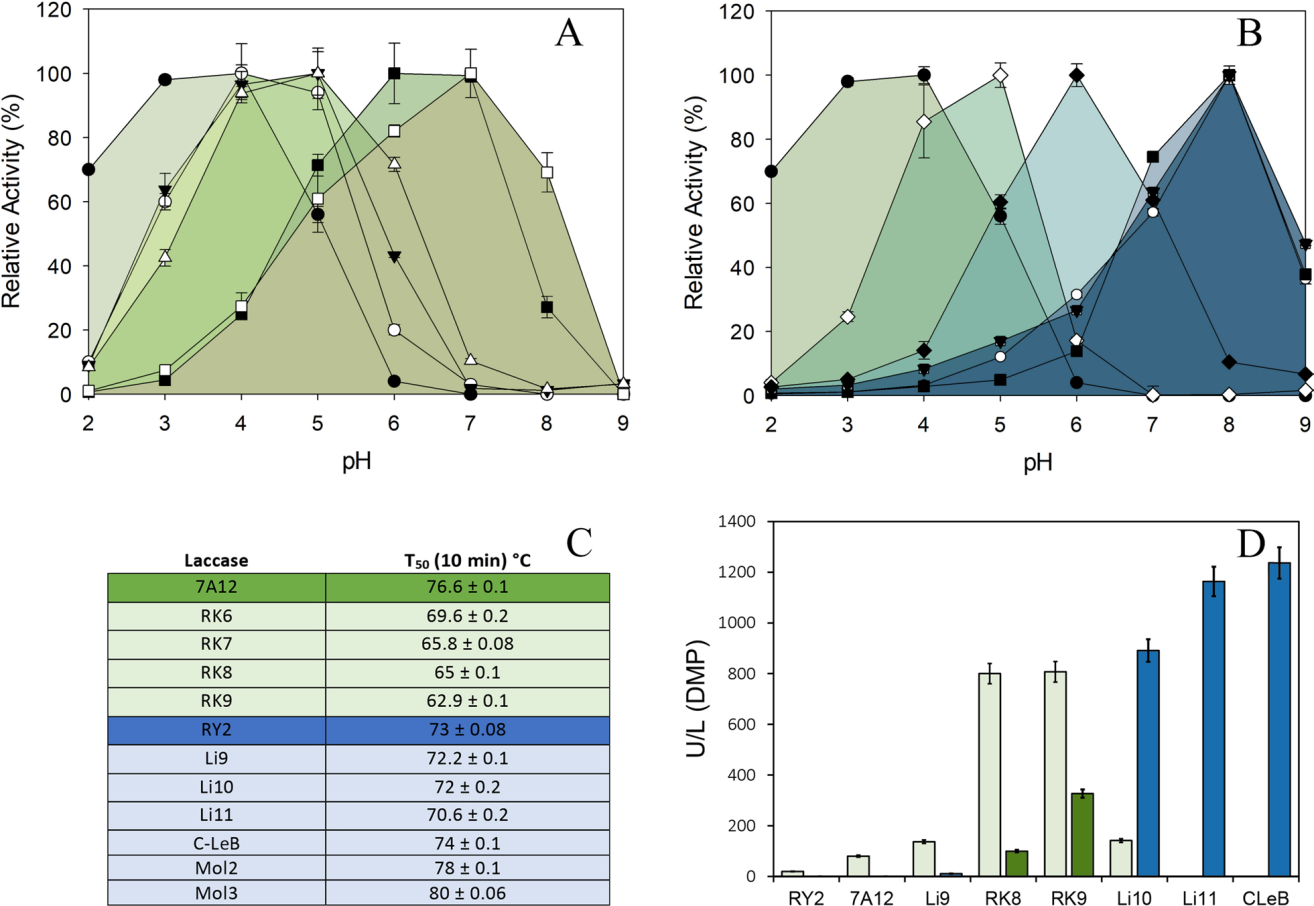
Shift of optimal pH and variations in thermal tolerance through enzyme engineering, and improvement of laccase activity. **a** First evolution pathway towards alkaliphilicity (7A12 lineage): parent 7A12, (white circles), RK6 (black inverted triangles), RK7 (white triangles), RK8 (black squares) and RK9 (white squares). **b** Second evolution pathway towards alkaliphilicity and thermophilicity (RY2 lineage): parent RY2 (white diamonds), Li9 (black diamonds), Li10 (white circles), Li11 (black squares), C-LeB (white squares), Mol2 (black circles) and Mol3 (black inverted triangles). Also shown is the activity profile of the original wild PM1 laccase (black circles). **c** T_50_ (10 min) of parent laccases 7A12 and RY2 and evolved variants selected during laccase directed evolution. **d** Activities detected in the liquid extracts of flask cultures of *S. cerevisiae* clones expressing the new alkaliphilic laccase variants from 7A12 (green) or RY2 (blue) lineages. Laccase activities were measured with DMP at pH 6 (light) and pH 8 (dark)

First, random mutagenesis of 7A12 laccase and screening of the mutant library resulted in the selection of RK6 variant, with mutation F396I, by improved activity at pH 6 (Fig. 2A). Conversely, the thermal tolerance of RK6 was decreased, as observed by the 7 °C drop in T_50_ (10 min) compared to parent laccase (Fig. 2C; Additional File 1: Fig. S1).

**Fig. 3:**
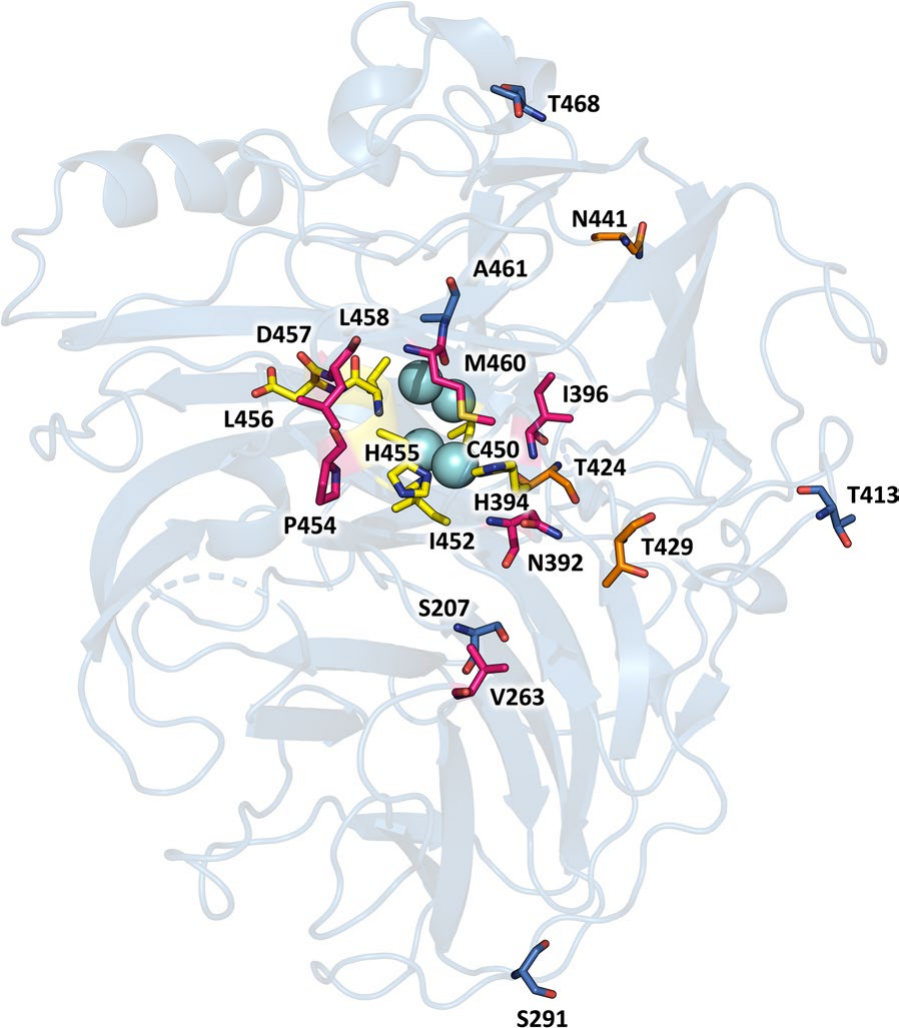
Molecular structure of Mol3 laccase. Cartoon representation of the 3D model structure of Mol3 laccase built with PyMol using 6H5Y.PDB as the template. Residues forming the first sphere of T1 catalytic copper are depicted as yellow sticks; residues mutated during the directed evolution of laccase towards alkaliphilicity are depicted as pink sticks; residues of difference between 7A12 and RY2 starting laccases are shown as blue sticks, and residues found to confer thermal stability to laccase in the bottom-up designing strategy are shown as orange sticks. Blue spheres represent the four catalytic coppers

Next, RK6 variant was subjected to saturation mutagenesis (SM) on Phe454 located in T1 site environment (Fig. 3; Additional File 1: Fig. S3). The new selected variant, RK7, containing mutation F454P, maintained pH 5 as the optimum with DMP, but showed 70% of activity at pH 6, and even 10% of activity at pH 7 (Fig. 2A). The T_50_ of the enzyme was again decreased by 4 °C (Fig. 2C; Additional File 1: Fig. S1).

A third evolution round consisted of SM of residue Phe392 in variant RK7. This residue delimits the substrate-binding pocket, and is located close to His394 ligand of T1 copper (Fig. 3; Additional File 1: S3) [26]. This time, we applied a stronger selective pressure during the screening by adding an assay with DMP at pH 8. Different mutations were found among the best selected clones (Additional File 1: Table S1). The best variant, named RK8 (with mutation F392N), was produced in flask, obtaining 100 U/L with DMP at pH 8 (Fig. 2D). The pH-dependent activity profile of RK8 was remarkably modified, showing maximum activity at pH 6–7 with DMP (Fig. 2A). Besides, RK8 laccase roughly retained the thermal tolerance of RK7 (Fig. 2C; Additional File 1: Fig. S1).

Finally, combinatorial saturation mutagenesis (CSM) of tripeptide Leu456-Asp457-Ala458 was completed in RK8 variant. Mutagenic primers were designed according to the most conserved residues found in the multiple alignment of laccases sensu stricto from 52 basidiomycete genomes [34] (Additional File 1: Fig. S2). Variant RK9 containing mutations L456I and A458L was selected among the clones of CSM library with improved activity on DMP at pH 8. The enzyme was produced in flask and characterized. While RK8 and RK9 variants maintained high activity with DMP at pH 6, the activity at pH 8 was enhanced threefold in the latter variant (Fig. 2C). The pH-dependent activity profile of RK9 laccase retained a clear maximum at pH 7, while exhibited 70% of activity at pH 8 (Fig. 2A). Conversely, a new decrease of 2 °C in T_50_ was observed in RK9 (Fig. 2C; Additional File 1: Fig. S1).

Considering the sharp decrease in thermal tolerance during the directed evolution of 7A12 laccase, with an overall decrease in T_50_ of 14 °C, from 76.6 °C in the starting point to 62.9 °C in RK9, and the need to obtain biocatalysts functional at alkaline pH and high temperature, a second laccase-directed evolution route was considered.

### Second evolutionary trajectory towards alkaliphilicity: RY2 lineage

In order to obtain alkaliphilic and thermophilic enzymes, we chose as a new starting point a robust engineered laccase, RY2, active and stable at high temperature and with maximum activity with DMP at pH 5, engineered [27] from yeast-expressed 7D5 laccase [32].

First, we took advantage of the mutations selected in 7A12 lineage to impart activity at alkaline pH to RY2 laccase. Since F454P mutation was already present in RY2 [27], we started by replacing Phe396 to Ile396 by site-directed mutagenesis, together with randomization of position 392 by SM (Fig. 1). Best selected variant, named Li9, with mutations F392N and F396I (Additional File 1: Table S2), was produced in flask and characterized. Its pH-dependent activity profile was completely different from that of parent RY2, with a maximum at pH 6 and 60% of activity at pH 7 with DMP (Fig. 2B). Importantly, the new variant retained the T_50_ of the parent laccase (Fig. 2C; Additional File 1: Fig. S1).

In a next evolution round, Li9 laccase was subjected to SM at Phe460 residue, located in the position of the fourth axial ligand of T1 copper in LRPLs (Fig. 3; Additional File 1: Fig. S3). Even though the mutagenic primers were designed with complete degeneracy of codons to force all possible substitutions of Phe460, only variants with Leu, Phe or Met in this position showed laccase activity. From this screening, we selected Li10 variant with mutation F460M. The activity with DMP at pH 8 detected in the liquid extracts of *S. cerevisiae* flask cultures were 90 times higher for Li10 than for Li9, and doubled the activity of RK9, the best alkaliphilic laccase obtained in the first linage (Fig. 2D). The optimal pH was clearly shifted, from maximum activity with DMP at pH 6 in Li9 laccase, to pH 8 in Li10 (and 40% activity at pH 9) (Fig. 2B). Moreover, Li10 retained the stability of parent RY2 laccase, showing a T_50_ around 72 °C (Fig. 2C; Additional File 1: Fig. S1).

Alkaliphilic Li10 variant was purified and its kinetic constants determined and compared with those of RY2 (Table 1). It was possible, for the first time, to determine the kinetic constants with DMP at pH 8, something totally impossible for the parent enzyme. Conversely, the catalytic efficiency for oxidation of ABTS at pH 3 was strongly diminished, due to the increase in *K*_m_. Turnover rate of Li10 variant for oxidation of DMP at pH 8 was similar to that of parent RY2 with DMP at pH 5, although the catalytic efficiency was reduced by the increment of *K*_m_ (Table 1).

**Table 1:** Kinetic constants of the alkaliphilic variants derived from RY2 laccase for the oxidation of different substrates

Laccase (mutations)	Substrate	pH	*k*_cat_ (s^−1^)	*K*_m_ (mM)	*k*_cat_/*K*_m_ (mM^−1^ s^−1^)
RY2 (parent)	ABTS	3	543 ± 17	0.01 ± 0.001	52,240 ± 569
	DMP	5	123 ± 6	0.26 ± 0.04	479 ± 76
	DMP	8	Undeterminable -		
Li10 (F392N, F396I, F460M)	ABTS	3	231 ± 5.6	1.3 ± 0.1	179 ± 16
	DMP	8	89 ± 6.2	20 ± 2.4	4 ± 0.6
	DMP	9	9.5 ± 0.1	1.1 ± 0.03	9 ± 0.1
Li11 (D263V, F392N, F396I, F460M)	ABTS	3	123 ± 5.5	0.5 ± 0.04	266 ± 26
	DMP	8	82 ± 7.6	1.5 ± 0.1	55 ± 6.7
	DMP	9	22 ± 1.3	0.3 ± 0.03	72 ± 8.5
	DMP	10	0.8 ± 0.03	0.027 ± 0.001	29 ± 1.5
C-LeB (D263V, F392N, F396I, A458L, F460M)	ABTS	3	136 ± 2.7	0.7 ± 0.04	200 ± 12
	DMP	8	42 ± 1.3	1.7 ± 0.1	25 ± 1.6
	DMP	9	26 ± 0.3	0.4 ± 0.01	61 ± 1.6
	DMP	10	2 ± 0.05	0.022 ± 0.002	95 ± 9
Mol3 (D263V, F392N, F396I, V424T, S429T, D441N A458L, F460M)	ABTS	3	113 ± 1.8	0.9 ± 0.02	125 ± 3.4
	DMP	8	63 ± 1.8	1.7 ± 0.2	37 ± 4.4
	DMP	9	16 ± 1.2	0.3 ± 0.02	53 ± 5.3
	DMP	10	0.7 ± 0.01	0.019 ± 0.001	37 ± 1.9

Kinetics for the oxidation of DMP as a lignin-derived phenol at alkaline pH and of ABTS (as a reference of laccase intrinsic acidic activity) by selected alkaliphilic and thermal-tolerant variants obtained by directed evolution of RY2 laccase. Mutations accumulated in the evolved variants are shown in brackets.

The high redox potential of parent laccase RY2 was maintained in the new alkaliphilic variant Li10 at pH 5.5 (Table 2). In addition, we determined the redox potentials of Li10 and of the later evolved alkaliphilic variant C-LeB at pH 8, to assess the possible influence of pH in the redox potential (E°) of T1 site (Table 2). We observed an increment of E° at pH 8 compared with pH 5.5. The E° of RY2 laccase could not be measured at pH 8.

**Table 2:** Redox potentials of alkaliphilic laccases

Laccase	*E*° (vs. NHE)	
	pH 5.45	pH 8.03
RY2	0.77 ± 0.01	Not determined
Li10	0.76 ± 0.02	1.12 ± 0.02
C-LeB	0.75 ± 0.01	1.11 ± 0.02

Next, assuming the important role of 263 residue in ligand positioning and binding, we randomized it in Li10 laccase to improve the affinity for lignin phenols at alkaline pH. The SM library was screened with DMP at pH 8 and guaiacol at pH 9, resulting in selection of three variants (with mutations D263V, D263G or D263A) with superior activity than Li10 laccase (Additional File 1: Table S1; Additional File 1: Fig. S4). The best one, Li11 (mutation D263V), was produced in *S. cerevisiae* flask cultures. Laccase activity detected in the liquid extracts raised from 800 U/L with DMP pH 8 for Li10, to 1200 U/L for Li11 (Fig. 2D). The latter variant showed similar optimum pH than Li10 for the oxidation of DMP (Fig. 2B) and guaiacol (Additional File 1: Fig. S5), and was nearly as stable as the rest of the variants of RY2 lineage (Fig. 2C; Additional File 1: Fig. S1).

Li11 laccase was purified and its kinetics constants measured (Table 1). The catalytic efficiency for DMP oxidation at pH 8 was improved 13-fold compared to Li10, due to a remarkably better *K*_m_. Furthermore, *K*_m_ and *k*_cat_ were improved for DMP oxidation at pH 9. Besides, it was possible to determine the kinetic constants with DMP at pH 10, something unbelievable for a basidiomycete laccase.

Thereafter, mimicking the directed evolution steps carried out in 7A12 lineage, we randomized the tripeptide LDA 456–457–458 in Li11 laccase. Different mutations were selected among the best clones of the CSM library screened with DMP and guaiacol at pH 8 and 9 (Additional File 1: Table S1). C-LeB variant (with mutation A458L) was chosen among those with the highest activity and produced in *S. cerevisiae* flask cultures. The new variant retained the thermal tolerance of the previous variants of RY2 linage (Fig. 2C; Additional File 1: Fig. S1). C-LeB also exhibited similar pH activity profiles than Li10 and Li11 with DMP (maximum activity at pH 8) and guaiacol (maximum activity at pH 9; Fig. 2B; Additional File 1: Fig. S5). Conversely, C-LeB displayed notably superior catalytic efficiency at pH 10, due to the significant higher *k*_cat_ and similar *K*_m_ (Table 1). Its redox potential was similar to that of Li10 (Table 2). The three purified alkaliphilic laccases, Li10, Li11 and C-LeB, were quite stable at pH 10–10.5 (Additional File 1: Fig. S6).

### Differences in tolerance to high temperatures of the two lineages of alkaliphilic laccases and design of new thermoresistant variants

Excluding the mutations introduced during this work, 7A12 and RY2 laccases solely differ in six residues: N/S207, T/S291, V/T413, D/E457, T/A461 and I/T468, respectively (Additional File 1: Table S2). In order to explain the loss of stability found during the evolution of the first lineage, we individually substituted the six amino acids of C-LeB by those present in 7A12. The six single-mutants S207N, S291T, T413V, D457E, A461T or T468I were transformed in *S. cerevisiae* and the corresponding clones cultivated in flasks for laccase production. In general, we detected minor differences in thermal tolerance or in laccase activity among the mutated variants (Table 3). The exceptions were the variant with mutation A461T, which exhibited an improvement in activity joined to a significant reduction of T_50_ compared to C-LeB, and S207N variant, whose low activity precluded the measurement of T_50_.

**Table 3:** Production and thermal stability of C-leB single mutants

Variant	Mutation	Activity (U/L)	T_50_ (10 min) °C
C-LeB	–	1812	74 ± 0.2
207	S207N	324	–
291	S291T	1765	74 ± 0.2
413	T413V	1850	74 ± 0.1
457	D457E	1798	75 ± 0.2
461	A461T	2230	69 ± 0.1
468	T468I	1839	74 ± 0.03

Activities (with ABTS, pH 3) detected in the liquid extracts of *S. cerevisiae* cells expressing C-LeB and its single-mutated variants cultured in flasks, and T_50_ (10 min) values of the laccase variants

**Fig. 4:**
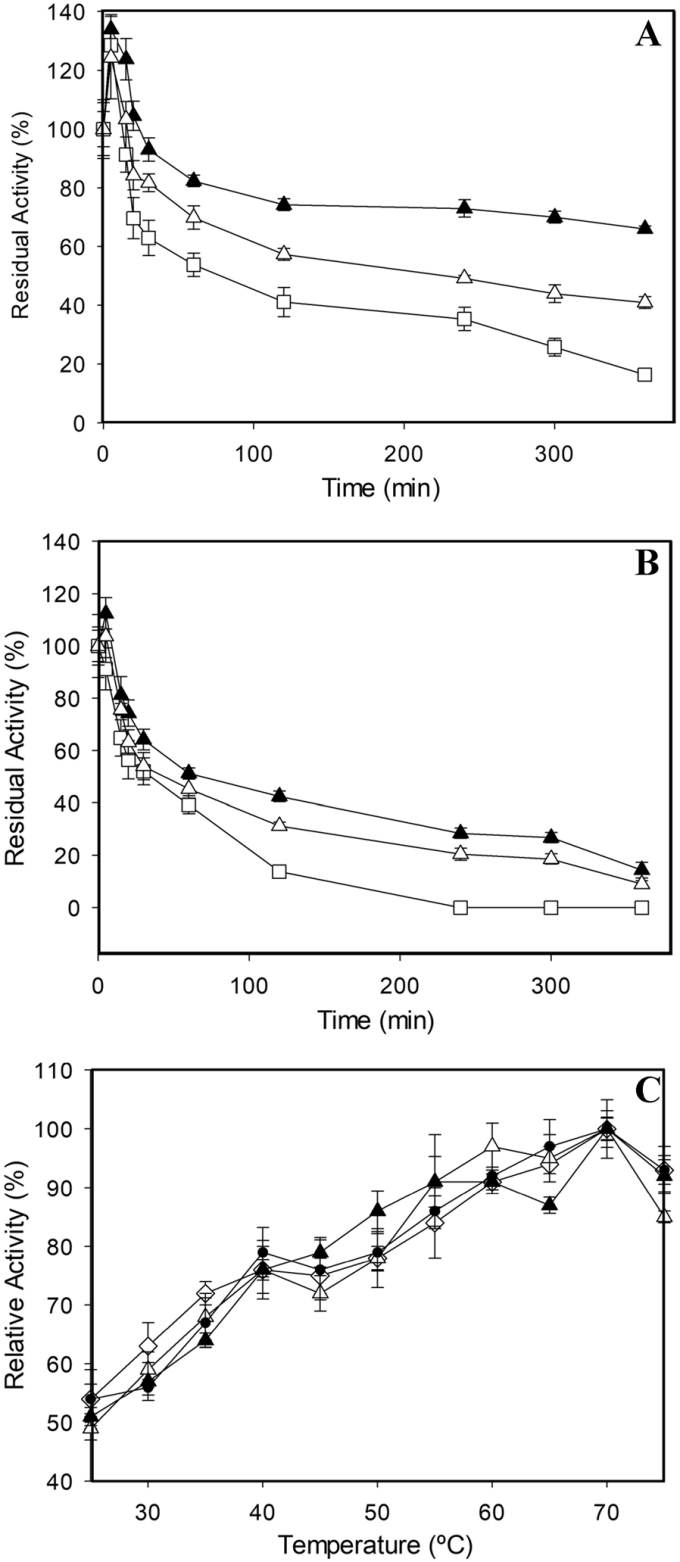
Thermostability and thermal activity of last variants engineered from RY2 laccase. Stabilities of pure laccase variants C-LeB (white squares), Mol2 (white triangles) and Mol3 (black triangles) after 6-h incubation at 60 °C (**a**) or 70 °C (**b**) and pH 7.5. Activities of same variants and parent RY2 (white diamonds) at different temperatures (**c**). Error bars indicate standard deviation for triplicates

With the aim of further improving the tolerance to high temperature of C-LeB alkaliphilic laccase, we simultaneously introduced six new mutations, V424T, N426S S429T, P430D, R439T and D441N (Fig. 3), based on reported data [35]. The resulting variant Mol2 was produced in flasks and compared with C-LeB. Its T_50_ was enhanced 4 °C (Table 4). Mol2 also displayed 20% superior residual activity at high temperatures after 6 h incubation (Fig. 4A, B). On the contrary, activity of Mol2 found in the liquid extracts of *S. cerevisiae* flask fermentations was lower (Table 4).

**Table 4:** Production and thermal tolerance of C-le B and Mol2 mutated variants

Laccase	Mutation	Activity (U/L)	T_50_ (10 min) °C
C-LeB	–	1812	74 ± 0.1
Mol2	–	1294	78 ± 0.1
*Top down*			
Mol2R-424	T424V	753	76 ± 0.07
Mol2R-426	S426N	1231	78 ± 0.04
Mol2R-429	T429S	531	76 ± 0.07
Mol2R-430	D430P	1522	78 ± 0.06
Mol2R-439	T439R	673	78 ± 0.08
Mol2R-441	N441D	1891	74 ± 0.09
*Bottom up*			
C-LeB-424	V424T	1500	72 ± 0.1
C-LeB-429	S429T	2791	75 ± 0.1
C-LeB-441	D441N	581	78 ± 0.1
C-LeB-DM1	V424T, S429T	1897	77 ± 0.09
C-LeB-DM2	V424T, D441N	1763	75 ± 0.04
C-LeB-DM3	S429T, D441N	1927	74 ± 0.1
Mol3	V424T, S429T, D441N	1180	80 ± 0.06

Laccase activities detected in the liquid extracts of *S. cerevisiae* clones cultured in flasks and T_50_ (10 min) values of C-leB and Mol2 laccases and their mutated variants (determined with ABTS, pH 3)

To assess the rationale behind the thermal stability of variant Mol2, we reverted the above mutations one by one in a top-down strategy. No differences in T_50_ were observed in S426N, P430D and R439T reverted mutants, whereas the reverted mutations T424V, T429S and N441D significantly reduced the thermal tolerance of Mol2 (Table 4). Therefore, the initial mutations corresponding to the last three were combined on C-LeB in a bottom-up strategy to assess possible positive epistatic interactions between them, and to further improve the thermal stability of the enzyme (Fig. 1). Simple (V424T, S429T and D441N), double (V424T, S429T; V424T, D441N; and S429T, D441N) and triple (V424T, S429T, D441N) mutants were designed, produced and characterized (Table 4). Simple and double C-LeB mutants showed equal (mutation D441N) or lower T_50_ than Mol2 laccase. Remarkably, the triple mutated variant (V424T, S429T, D441N), named Mol3, exhibited a 2 °C-improved T_50_ compared to Mol2, with similar laccase activity detected in the liquid culture.

Variant Mol3 was purified, characterized and compared with Mol2 and C-LeB. The three laccase variants of RY2 lineage are thermoactive, showing same maximum activity at 70 °C as the parent laccase (Fig. 4C). Besides, variant Mol3 was remarkably more stable, as observed by the higher residual activities after several hours of incubation at high temperatures (Fig. 4A, B). The half-lives of Mol3 at 60 °C and 70 °C doubled those of the thermotolerant variant Mol2, surpassed by eightfold and threefold the half-lives of C-LeB at 60 °C and 70 °C, and were up to fivefold the half-lives of RY2 (Table 5). The kinetic parameters of the thermostable laccase Mol3 were similar to those of C-LeB variant, with a slight increment in catalytic efficiency for DMP oxidation at pH 8, and the opposite effect at pH 10 (Table 1).

**Table 5:** Half-lives of RY2 parent laccase and its last evolved alkaliphilic and thermophilic variants

*T* (°C)	*t*_1/2_ (h)			
	RY2	C-LeB	Mol2	Mol3
60 °C	2.0	1.3	5.5	10.2
70 °C	0.4	0.6	0.8	1.5

Residual activities of the pure enzymes were determined with ABTS, pH 3

Finally, in order to improve the production of the last evolved extremophilic variant Mol3, that was diminished compared to C-LeB (Table 4), the CDS of Mol3 was fused to an optimized signal peptide based on the α-factor pre-proleader, which has been recently designed to enhance the secretion of fungal enzymes by the yeast [29]. Production of Mol3 laccase with the α_OPT_ leader (Additional File 1: Fig. S7) doubled the levels obtained with the evolved α_9H2_ leader [27] that was used so far for the rest of the variants developed in this work due to its preceding demonstrated capability to increase laccase secretion by the yeast [29]. In addition, *Komagataella pastoris* has been used as host for scaling up the production of some of the extremophilic variants evaluated in the application tests (see below).

### Enzyme application tests

The tailor-made extremophilic laccases were assayed as biocatalysts in three applications: (1) depolymerization of kraft lignins, (2) delignification and bleaching of kraft pulps and (3) production of fibreboards.

**Fig. 5:**
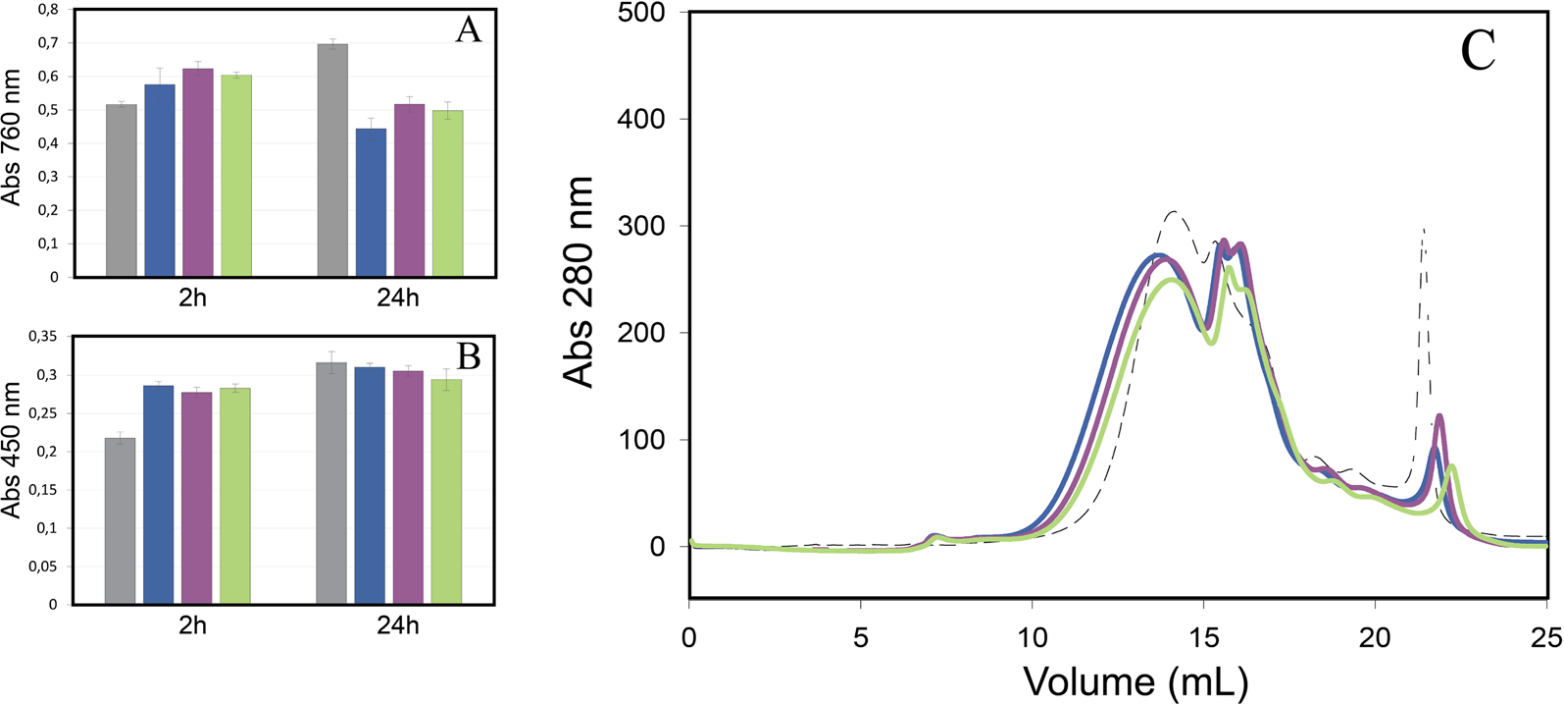
Treatment of eucalyptus kraft lignin with alkaliphilic laccases at pH 10. Content in phenolic (**a**) and carbonylic groups (**b**) in control (grey) and treated eucalyptus kraft lignin with laccases Li10 (blue), Li11 (purple) and C-LeB (green). Each point represents the average of three independent experiments ± standard deviation. SEC profile of eucalyptus kraft lignin before (dotted line) and after treatment with Li10 (blue), Li11 (purple) and C-LeB (green) laccases for 24 h (**c**)

**Fig. 6:**
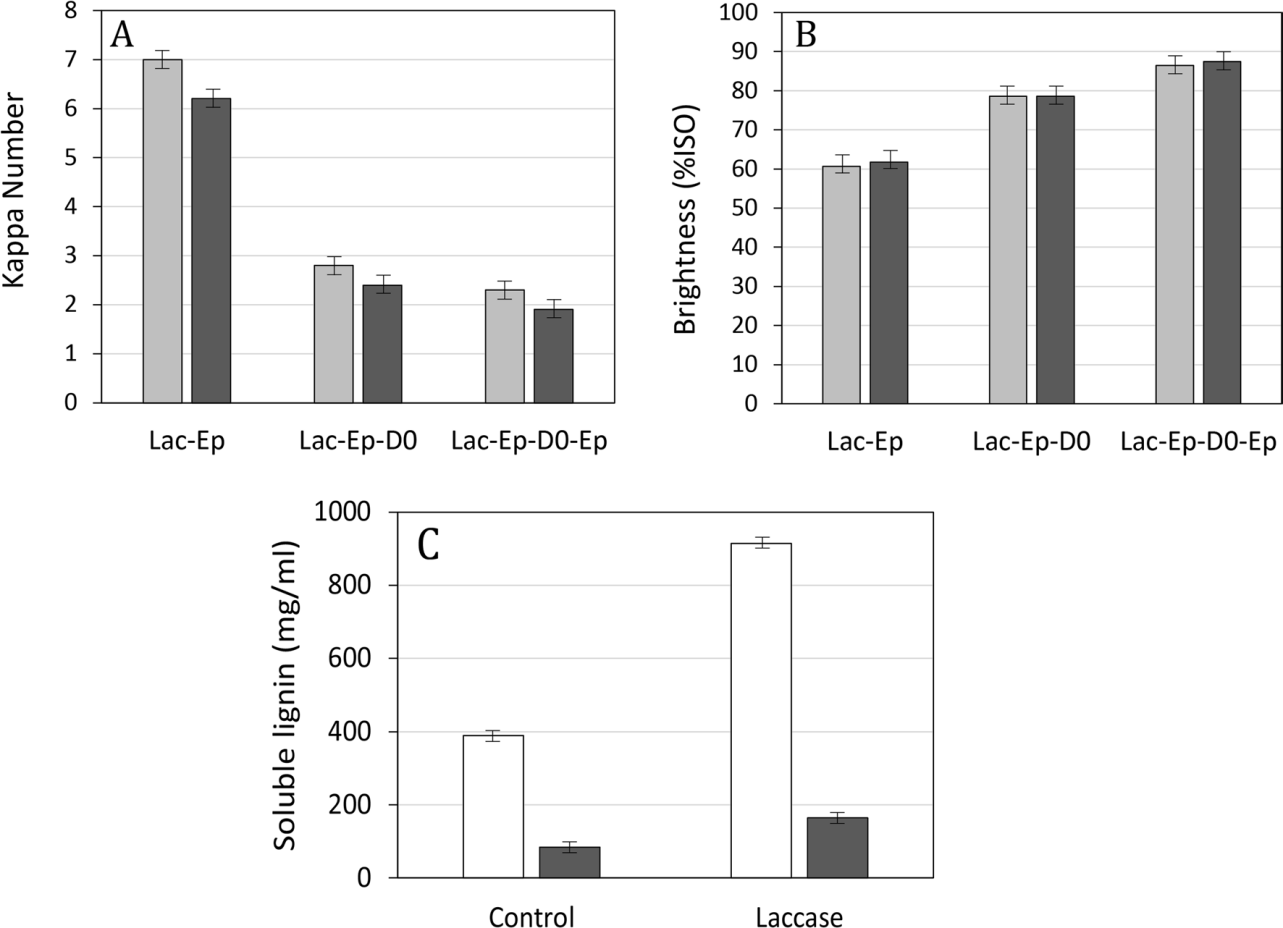
Bleaching of hardwood kraft pulp with alkaliphilic laccase. Evolution of kappa number (**a**) and pulp brightness (**b**) in control hardwood kraft pulp (light grey) or after Li10 laccase stage (dark grey) followed by Ep, D0, and Ep stages. Soluble lignin (**c**) in the effluent generated from hardwood kraft pulp before (white) and after Ep stage (grey). Error bars indicate standard deviation for duplicates

1. We assayed the capability of the alkaliphilic laccases Li10, Li11 and C-LeB to catalyse the oxidation at pH 10 of a kraft lignin extracted from the black liquors of a eucalyptus kraft pulp mill. We determined the phenolic and carbonylic contents in the enzymatically treated lignin samples and control lignin (treated under same conditions without enzyme), and observed a strong modification of these functional groups upon oxidation by the three enzymes (Fig. 5A). The content in phenolic OH groups increased after 2 h of enzymatic treatment, and then decreased after 24 h. The content in carbonyl groups increased after 2 h of laccase treatment, although no significant changes were found after 24 h compared to control lignin (Fig. 5B). Besides, the molecular weight (Mw) distribution profile of kraft lignin was also significantly modified after 24 h of reaction with laccase (Fig. 5C), with some differences observed after oxidation with the different laccase variants.
2. Laccase Li10 in the presence of methyl syringate as redox mediator was tested as a pre-bleaching stage of hardwood kraft pulp after oxygen delignification. Once enzymatically treated, the pulp was subjected to the bleaching sequence Ep D0 Ep (Ep: alkaline extraction in the presence of hydrogen peroxide; D0: chlorine dioxide). Li10 laccase was able to delignify the kraft pulp as evidenced by the reduction in kappa number after L stage compared to control kraft pulp (treated under same conditions without enzyme), followed either by Ep, Ep D0 or Ep D0 Ep (Fig. 6A). The enzyme also increased pulp brightness from 60.7% ISO of control lignin after Ep or 86.5% ISO after Ep D0 Ep, to 61.8% ISO after L Ep or 87.5% ISO after L Ep D0 Ep (Fig. 6B). Laccase stage also produced a notable increment of the lignin released in the effluent before (from 390 to 915 mg/ml) or after Ep stage (from 85 to 165 mg/ml) (Fig. 6C).

Furthermore, the extremozyme-aided bleaching sequence (L Ep D Ep) was compared to the industrial D Ep D D sequence used for bleaching the hardwood kraft pulp. Although the L Ep stages seemed to be less efficient than the D Ep ones in terms of brightness, the final brightness after the complete bleaching sequence was comparable: 88.6% ISO for L Ep D Ep versus 88.8% ISO for D Ep DD. Because the final pulp brightness was reached with the L Ep D Ep sequence, a ClO_2_ saving of 11% could be envisaged by the mill. Finally, it was possible to reach very high level of brightness (91% ISO) by adding a final D stage (0.72% ClO_2_) to the laccase-assisted L Ep D Ep sequence, with a limited ClO_2_ consumption (95%).

**Fig. 7:**
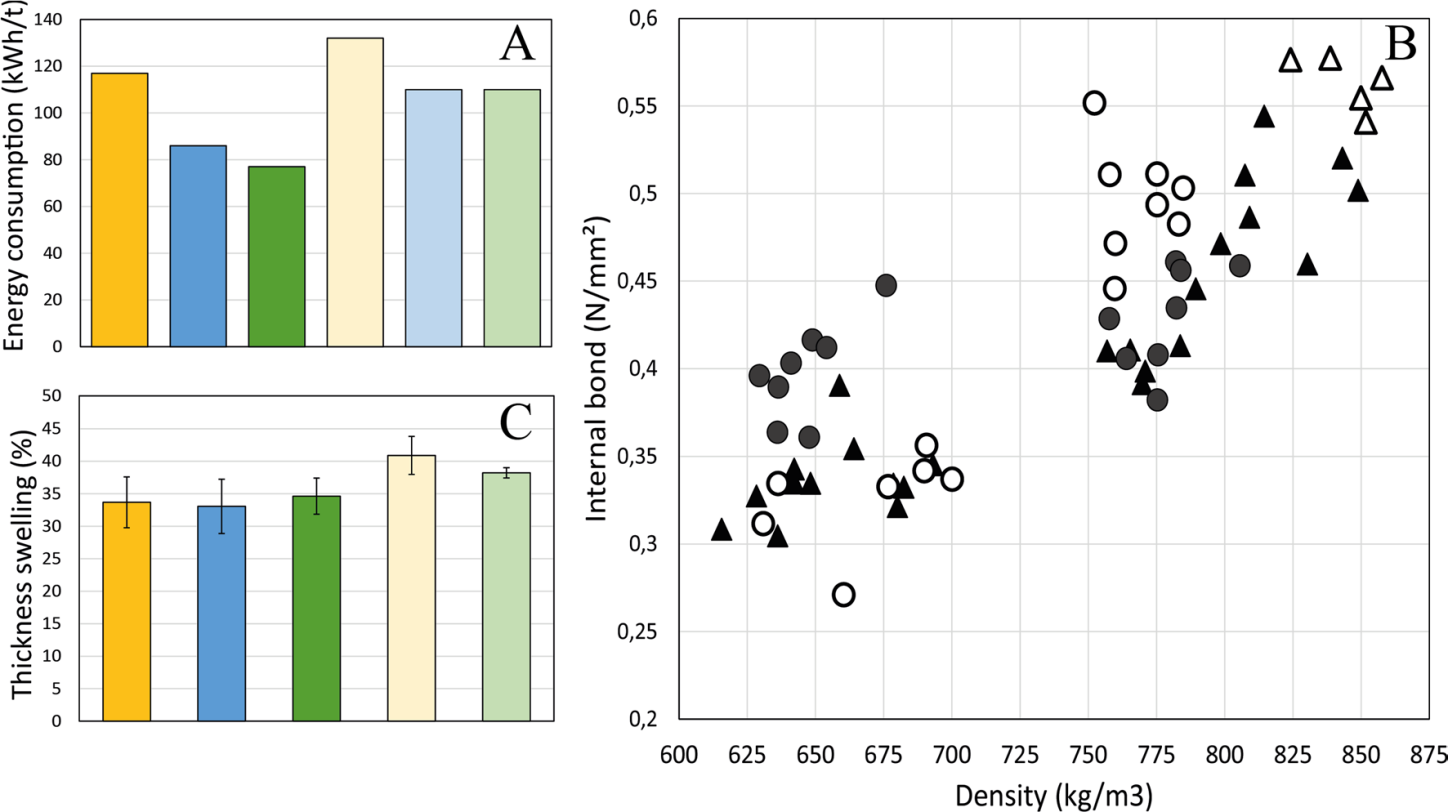
Treatment of wood chips for MDF manufacture with addition of laccase. **a** Energy consumed during defibering of wood chips at different conditions: (i) without compression screw: raw material without immersion (dark yellow), control with 120′ immersion + 20′ steaming (dark blue) and laccase plus 120′ immersion + 20′ steaming (dark green); or (ii) with compression screw: raw material without immersion (light yellow), control with 60 immersion + 10′ steaming (light blue) and laccase plus 60′ immersion + 10′ steaming (light green). **b** Internal bond as a function of density in MDF produced with fibres obtained applying enzymatic treatment with (white circles) or without compression screw (white triangles). Controls with and without compression screw are depicted as black circles and black triangles, respectively. **c** Swelling in water as a function of density of MDF produced with fibres obtained applying same conditions shown in **a**

3. Wood chips were treated with Li9 laccase prior to defibering during the production of medium-density fibreboards, using different processing conditions. The different processing conditions assayed strongly determined the energy consumed, both in the enzymatically treated wood chips and in the control ones (Fig. 7A). With compression screw and 10 min steaming, chip defibering required 132 kWh/t, whereas defibering of chips with water immersion only and no compression screw, but a longer steaming time (20 min) required less energy (86 kWh/t). The resulting fibres were used to produce MDF boards and their performances in terms of internal bonding (Fig. 7B) and water swelling (Fig. 7C) were determined.

Internal bond increased with density, the higher density, the higher their interactions. Limited differences were observed in the fibre bonding potential due to the enzymatic treatment, although a slight positive effect could be found in the boards with higher densities.

As regards water swelling, it was affected mainly by density, the denser the board, the higher water swelling. The defibering conditions had a strong impact on this property. Increasing steaming time resulted in higher water swelling as observed for all densities. This might be due to higher modification of lignin or hemicelluloses. In addition, boards treated with Li9 laccase showed reduced water swelling, precisely in the chips longer steamed.

## Discussion

### Laccase engineering towards alkaliphilicity

Wild fungal laccases commonly show maximum activities at pH 2–3 with ABTS and pH 3–5 for oxidation of phenolic compounds [36]. In fact, the wild fungal laccase, PM1L, has maximum activities at pH 2 with ABTS and 3–4 with DMP [33], while 7A12 shows maximum activities at pH 3 with ABTS [32] and pH 4 with DMP (Fig. 2A). Both enzymes showed the typical pH-dependent bell-shaped activity profile for oxidation of phenolic compounds. This is explained by two opposite effects: the increment of enzyme activity with increasing pH, due to the decrease in the reduction potential of phenols, and the decrease of laccase activity at high pH, due to the inhibition of oxygen reduction in TNC by hydroxide anions (OH-) [37].

Phe396 is highly conserved and seems to contribute to the redox potential of the HRPL together with other residues of T1 copper environment [38]. Phe396 is contiguous to His397 ligand of T2 copper, and acts as a bridge connecting the T1 site and TNC via Pro395 and His394 (T1 copper ligand) (Additional File 1: Fig. S3). Mutation F396I was reported as responsible for remarkable improvement of laccase activity at neutral pH during the directed evolution of another variant of PM1L [39]. Remarkably, when mutation F396I was introduced in RY2 lineage (Li9 variant), the thermal resistance of the enzyme was preserved, in contrast to the reduction of T_50_ by 7 °C observed in 7A12 linage (RK6 variant; Fig. 2C; Additional File 1: Fig. S1).

Substitutions of Phe454 had been reported to improve catalytic activity, highlighting this position as a hotspot for laccase engineering, although the stability of the enzyme can be jeopardized. Replacement of Phe454 by serine increases laccase activity but produces a loss of stability, that is explained by reduced number of hydrogen bonding [27, 40]. Other polar residues like threonine in this position heavily diminish the enzyme stability at high temperature, while basic histidine or non-polar proline, especially the latter, scarcely affect protein stability. Mutation F454P seems to be also responsible for shifting the laccase pH profile, improving activity at pH 5–6 [27]. In the first case, mutation F454P did not affect enzyme thermal tolerance, whereas here this same mutation decreased by 4 °C the T_50_ of RK7 compared to RK6.

A high number of active clones were found during the screening of the mutant libraries obtained by SM of Phe392 in both laccase linages, in agreement with the high variability of amino acids occupying this position in fungal laccases [26]. Mutations F392Y and F392T repeatedly appeared in the two evolutionary trajectories among the clones with better activities with DMP at pH 6–8 (Additional File 1: Table S1). Mutation F392N coincided as the best one in both laccase lineages (variants RK9 and Li9). Mutations F392N and F392T were also selected in a previous study, proving to promote the interaction of phenolic substrates in the active site by opening the enzyme pocket, and resulting in the shift of activity towards more neutral pH [26]. Other mutations in this position seem to modify the binding site anchor [41].

The high number of non-functional clones found during screening of the SM library over Phe460, and the repeatedly presence of Phe, Leu or Met in the active ones, reassembled the exclusive occurrence of these three amino acids during natural evolution of laccases [42, 43]. In alkaline medium, the inhibition effect of OH- in the reduction of oxygen at the TNC favour fungal laccases to have optimum activities at acidic pH [37]. The negative effect in enzyme activity at high pH is more pronounced in HRPLs where the rate-limiting oxidation step is the intramolecular electron transfer between T1 and TNC [44], which is interrupted by the presence of OH-. In contrast, the electron transfer from the substrate to T1 copper constitutes the rate-limiting step in LRPLs [44]. Indeed, bacterial LRPLs commonly show more alkaline activity profiles.

Replacement of Phe by Leu and vice versa in the position of the fourth axial ligand was described in other laccases as non-altering the redox potential of T1 site [22, 44]. This result can be explained by the fact that neither Leu nor Phe coordinate the T1 Cu and, therefore, do not directly donate charge to the copper. On the contrary, replacement of Leu or Phe by Met had a significant effect on the redox potential (E^o^) of laccase, reducing the E° of the T1 site when Met is introduced, and augmenting it when Leu or Phe replace Met [44]. By contrast, Li10 and C-LeB laccases developed here, which are active on lignin phenols at pH 8–10, hold a Met in this position due to the presence of mutation F460M, but conserve the high redox potential of parent laccase RY2 (with a Phe in this position). On the other hand, the observed increment in their redox potentials with pH, agrees with reported E° increase in *T. villosa* laccase at pH 8 [44]. In that case, the direct correlation of increasing E° with pH was more pronounced when Phe was replaced by a Met in the position of the fourth axial ligand. This type of pH-positive correlation between the oxidation at higher pH and laccase E° has been observed in other studies, and it has been hypothesized that increase in E° at the T1 site may overcome the loss of activity at higher pH values [22].

The loss of affinity for DMP at alkaline pH observed in Li10 variant may be related to changes in the protonation state of the residues of the catalytic pocket, from more positively to negatively charged with increasing pH, which might disturb the positioning and binding of the substrate [26]. Besides, replacement of hydrophobic Phe392 delimiting the substrate-binding pocket by polar Asn in Li10 may also alter the substrate–protein interaction [26]. Also, deprotonation of lignin phenolic compounds at alkaline pH [45] might influence substrate binding.

During the laccase engineering campaign resulting in RY2, Asn263 located in the entrance of the substrate-binding pocket was mutated to Asp263 with the objective of improving the affinity for aniline at acid pH [27]. This aspartate residue stabilized the anilinium cation in the active site and favoured the electron transfer at acidic pH [46]. The mutations selected here in position 263 demonstrated that non-charged, non-polar and small amino acids (Val, Ala, Gly) are preferred for the oxidation of phenolic substrates at pH ≥ 8 (Additional File 1: Fig. S4). The remarkably better *K*_m_ for DMP at pH 8 in Li11 laccase highlights the role of Val263 to facilitate accessibility of the substrate to the enzyme binding pocket. It is worth mentioning that Asn263, which is the most conserved amino acid in this position in naturally evolved laccases [46], was not found among the most active clones during the screening.

Mutagenesis on LEA/LDA was performed based on the most conserved residues in laccases sensu stricto from 52 basidiomycete species [34]. The high number of non-functional clones detected during the screening suggested that these positions play a crucial role in laccase activity. In fact, CSM libraries from the two laccase lineages coincided in a majority of active clones holding the consensus amino acid residues. The second position (457) was in all cases occupied by a negatively charged acidic residue (Asp or Glu) (Additional File 1: Table S1), whereas the first and third positions tolerated more variability. By contrast to what happens in the 7A12 lineage (RK9 laccase), mutation A458L did not changed the pH profile in C-LeB, although it had a strong positive effect on the catalytic efficiency of the enzyme at pH 10 (Table 1).

LEA/LDA tripeptide, located in the binding pocket, has been conserved during the evolution of basidiomycete HRPLs, whereas VSG tripeptide appears to be conserved in LRPLs [22]. However, no differences in the redox potential of *Rhizoctonia solani* basidiomycete laccase were found after switching LEA with VSG (and vice versa* in Myceliophthora thermophila* laccase), although changes in the *k*_cat_ and *K*_m_ and optimum pH were observed [22]. The latter could be attributed to electrostatic and steric hindrances to substrate docking [26]. Similarly, we found no changes in E° after mutating Ala458 in Li10 laccase (C-LeB variant), although we found a significant improvement in the kinetics constants at alkaline pH. This work confirms how the structural rearrangement surrounding the T1 Cu site is crucial to modify pH-dependent activity of laccase [22, 39, 44, 47, 48].

The design of fungal laccases with alkaline activity is a long-sought challenge to expand their potential as industrial biocatalysts. Some studies have reported the design of fungal laccases with more neutral-to-alkaline activities [39, 41, 48–51]. However, most of them show enhanced activities at pH 8 or lower [39, 41, 49, 50], whereas only one reporting similar kinetic parameters at pH > 8, corresponds to a medium-redox potential enzyme from Ascomycota [48]. In addition, although other studies have reported basidiomycete laccase activities above pH 8 [51], we achieved to obtain laccases with improved catalytic activities at pH 8–10 and report for the first time kinetic parameters for DMP oxidation at pH 10. Finally, the new alkaliphilic laccase variants maintain the high redox potential of the parent type.

### Thermal tolerance of the alkaliphilic laccases

The variants successively evolved from 7A12 laccase, progressively lost tolerance to high temperatures, resulting in a total decrement of 14 °C in T_50_ in the final alkaliphilic variant RK9. Quite the opposite, thermal tolerance was preserved in all intermediate variants of RY2 laccase, until the final alkaliphilic variant C-LeB, even when equivalent mutations were introduced in this second lineage. This fact points out the dependence of the outcome of these mutations on the genetic background of the two starting laccases, that allow to accumulate mutations in RY2 without jeopardizing stability, but not in 7A12.

There is an inherent trade-off between the rigidity necessary for enzyme stability and the flexibility required for enzyme activity. Similarly, during directed evolution, it is common to find stability losses when improving enzyme activity [52]. Mutation A461T was selected during the first directed evolution campaign of PM1 laccase due to an increment on enzyme activity [53]. DNA shuffling of this enzyme with another HRPL from *P. cinnabarinus* also developed in *S. cerevisiae* [54] rendered a set of new laccase variants, of which 7A12 conserved mutation A461T, but 7D5 did not [40] and, consequently, neither did RY2 laccase developed from 7D5 [27]. Presumably, Thr461 improved laccase activity but it produced a destabilizing effect on the enzyme, that is put in evidence when new mutations are introduced in the T1 Cu environment. Also, it seems that the highly conserved Ala461 (Fig. 3) adjacent to the “four-axial ligand” plays a crucial role in the kinetic stability of laccase.

The set of 6 mutations introduced in C-LeB to obtain Mol2 variant were selected as responsible for enhanced thermal stability of another PM1L variant during DNA shuffling with other basidiomycete laccase [35]. We prove here that of this set of mutations only three, V424T, S429T, D441N, increase enzyme thermal stability (Mol3 variant). The remaining three mutations, N426S, P430D, and R439T, do not exert a positive effect on the stability of laccase. Most likely, they were selected along with the beneficial ones during the recombination event [35] due to their proximity in the protein sequence. We also demonstrate the non-additive but epistatic interaction of mutations V424T, S429T, D441N, resulting in a positive effect on the thermal stability of the enzyme compared to their effect alone (or in pairs). The prevalence of epistasis in enzyme adaptive evolution, in particular positive epistasis, has been fully demonstrated [25, 26, 55]. Interestingly, mutations that are neutral or negative on the wild-type background, can become beneficial at a later stage in the evolutionary trajectory. The beneficial substitutions selected here are not close in the protein folding. However, epistasis is not only associated with direct interaction between residues, it is also caused by long-range indirect interactions between mutations [25, 56].

### Enzyme application tests

Enzymatic depolymerization of kraft lignins into phenolic platform chemicals and polyols embodies one of the potential breakthrough applications for valorization of kraft lignins whose recovery in pulp mills was 265,000 t in 2018 [57] and it is expected to increase in the coming years. Moreover, extracting part of the lignin from the black liquor to be transformed in bio-based products will help to de-bottleneck the recovery boiler in the kraft process, resulting in an increase of pulp production capacity.

Kraft lignins are characterized by the high content in free phenolic OH groups caused by the breakdown of aryl-ether bonds during kraft pulping [58]. This greatly favours the oxidation of kraft lignin by laccase to form phenoxyl radicals. Oxidation of lignin units by laccase leads also to the oxidation of side-chains and the production of quinoid structures [59], thus explaining the observed increment of carbonyl groups after oxidation. However, phenoxyl radicals have strong tendency to couple, giving rise to condensation reactions that result in the formation of new ether and C–C linkages [60] after laccase treatment. Thus, repolymerization reactions counteract the depolymerization of lignin produced by the enzyme [61]. Although the balance between cleavage and condensation reactions depend on multiple factors such as the type and concentration of the laccase used, or the pH and temperature of the reaction [62], repolymerization of lignin products commonly prevails in one-pot reactions in the lab, precluding direct measurement of enzymatic depolymerization of lignin. Here, lignin oxidation was evidenced by the increment in carbonyl content after 2 h of laccase treatment, whereas the increment of free phenolic groups suggested bond cleavage and, therefore, lignin depolymerization. Thereafter, the decrease in the phenolic content [63] and some increased in the Mw distribution of lignin after 24 h of laccase treatment correlate with the occurrence of non-enzymatic repolymerization reactions, although some low-Mw peaks were also found. Direct demonstration of enzymatic depolymerization of lignin can only be confirmed using advanced technologies, such as METNIN™ lignin refining technology, that avoid repolymerization of lignin products by combining enzymatic depolymerization of lignin at alkaline pH by a bacterial laccase with a cascading membrane operation [64].

Laccases can also aid delignification and bleaching of kraft pulps in the presence of redox mediators [65, 66]. The use of alkaliphilic and thermophilic laccases can provide the advantage of smoother integration in the industrial bleaching sequences, as shown here. The integration of an extremozyme-mediator pre-bleaching stage in the bleaching sequence significantly improved the delignification and bleaching of hardwood kraft pulp. This allows to either eliminate one D stage, with notable ClO_2_ savings and reduced environmental impact, or attain higher brightness levels than reference mill, even with limited ClO_2_ (95%) consumption.

Finally, enzymatic treatment of wood chips might facilitate fibre separation from the wooden matrix during defibering and/or pre-activating the fibre surface. Here, we prove that the impregnation of wood chips with an engineered laccase before fibre forming reduces the electrical energy consumed during defibering. In addition, the laccase treatment reduced the water swelling of the fibreboards, especially in chips longer steamed which is highly beneficial because the latter present the highest values of water swelling.

## Methods

### Construction of mutant libraries

Laccases 7A12 [32] and RY2 [27] developed and expressed in *S. cerevisiae* were fused to the evolved alpha-factor pre-proleader α_9H2_ [27] and used as starting points of the evolution pathways.

Error-prone PCR was carried out under described conditions [54], using Mutazyme II DNA polymerase at low mutagenic rate (GeneMorph II Random Mutagenesis kit). Primers Ext-pJRoC30-F sense and RMLC Ext-pJRoC30-R (Additional File 1: Table S3) were designed to generate overhangs of over 20 bp homologous to the ends of the linear vector in the PCR products to facilitate the in vivo cloning in the yeast [67].

Site directed mutagenesis of F396I was performed at PCR described conditions [46]. Same procedure was used for the directed mutagenesis in V413, S291, D457, A461, T468 and for all the variants developed for increasing the thermal stability of C-LeB laccase (Top-Down and Bottom up mutants). All the primers used in these assays are depicted in Additional File 1: Table S3.

Saturation mutagenesis on Phe454 was carried out using primers 454SM-Fw sense and 454SM-Rv antisense combined with Ext-pJRoC30-F sense and RMLC Ext-pJRoC30-R, respectively (Additional File 1: Table S3). For 50 µL reaction, 5 µL buffer Phusion, 3 µL DMSO, 1 mM dNTPs mix, 2.5 µL each primer (0.25 µm), 1 µL Phusion polymerase and 100 ng of DNA template were added. PCR was carried out under the following conditions: 95 ℃ (2 min), 1 cycle; 94 ℃ (30 s), 55 ℃ (30 s), 74 ℃ (2 min), 28 cycles; and 74 ℃ (10 min), 1 cycle. Same procedure was used for the saturation mutagenesis of F392 and F460 using 392SM-Fw sense and 392SM-Rv antisense, and 460 Li9SM-Fw sense and 460Li9-Rv antisense oligos, respectively. NNK degeneracy of codons was used.

CSM libraries on residues 456–457–458 were performed using mutagenic primers LEA-Fw sense and LEA-Rv antisense (Additional File 1: Table S3). Degeneracy of the codons enabled the replacement of LEA/LDA by the preferred amino acid residues found during the natural evolution of basidiomycete laccases (Additional File 1: Fig. S2), that is NTS VMS VNA. PCR conditions were the same as used for SM.

The optimized α_OPT_ leader [29] replaced the α_9H2_ leader for the production of Mol3 variant to improve enzyme secretion *in S. cerevisiae.*

In each evolution round, PCR products were purified and 400 ng mixed with 100 ng of linearized pJRoC30 vector, and transformed into competent cells of protease-deficient *S. cerevisiae* BJ5465 strain using a yeast transformation kit (Sigma). Transformed cells were plated on SC dropout plates and incubated for 2 days at 28 °C. Colonies containing the whole autonomously replicating vector were selected and cultivated in microplate fermentations [54].

### Screening of mutant libraries

Up to 2800 *S. cerevisiae* clones of the epPCR library were screened for laccase activity. For saturation mutagenesis libraries at least 145 clones were screened for 99% coverage of all possible substitutions. In the case of CSM 456–457–458 library, activities of 3450 clones were screened to attain 95% coverage with NTS VMS VNA possible substitutions.

Individual clones from the mutant libraries and the corresponding parent laccases were picked and cultured in 50 µL of minimal medium in sterile 96-well plates [53]. The plates were sealed and incubated at 30 °C in 80% humidity with 180 rpm agitation. After 48 h, 160 µL of expression medium [54] was added and the plates were incubated for 24 h. Then, plates were centrifuged (6000 rpm, 4 °C, 5 min) and 20 µL supernatant aliquots were transferred to two replica plates using a liquid-handling robot to carry out the enzymatic reactions [54].

Laccase activities of mutant libraries were screened with DMP and guaiacol. The selective pressure was intensified through the evolution pathway by increasing the pH of the assays from pH 6 to pH 8 and 9. In addition, oxidation of 3 mM ABTS at pH 3 in citrate phosphate buffer was used to monitor the intrinsic acidic activity of laccase along the successive evolution rounds. Enzymatic reactions were started by adding 180 µL of substrate solution (3 mM ABTS in 50 mM citrate phosphate buffer pH 3, 3 mM DMP in 50 mM Britton-Robinson buffer pH 6 and 8, and 9 mM guaiacol in 50 mM Britton-Robinson buffer pH 9). Laccase activity in the wells were measured in a Spectra max plus 384 plate reader (Molecular Devices) by monitoring the oxidation of the substrate at the maximum absorbance of the oxidized product (ABTS: 418 nm, DMP: 469 nm, guaiacol: 470 nm) in kinetic mode. Best variant selected by improved oxidation of lignin phenols in each directed evolution round was used as parent for the next round.

### Flask production and purification of selected laccases

The *S. cerevisiae* clones expressing selected laccase variants were grown in duplicate, in 100 mL or 1L flasks with 30 mL or 300 mL expression medium, respectively, containing 1 M CuSO_4_ and 3% ethanol [54]. Laccase activity secreted in the liquid cultures was monitored over time by measuring the oxidation of 3 mM ABTS in 50 mM citrate phosphate buffer pH 3, 3 mM DMP 100 mM sodium phosphate buffer (pH 6 and 8) and 9 mM guaiacol in 100 mM Britton-Robinson buffer pH 9 with UV-1900 Shimadzu spectrophotometer. After 3d fermentation, cultures were centrifuged, filtered and concentrated [27].

Purification of selected enzyme variants by high-pressure liquid chromatography (HPLC) was carried out in 3 chromatographic steps: two anion exchange steps using a HiPrep-QFF 16/10 column in a 100 mL gradient of 0–40% elution buffer, and a MonoQ-HR 5/50 column in a 30 mL gradient of 0–25% elution buffer, followed by size exclusion chromatography with a Superdex75. Fractions containing laccase activity (with 3 mM ABTS in 50 mM citrate phosphate pH 3) were pooled, dialyzed in Tris–HCl pH 7 and concentrated after each chromatographic step. Enzyme purification was confirmed by the electrophoretic mobility of the proteins in SDS-PAGE (12% acrylamide) stained with Coomassie Brilliant blue.

### Enzyme characteristics

Characterization of pure and non-purified (crude) laccases was carried out in 96-well plates with enzyme aliquots of 0.1 U/mL activity (measured with 3 mM ABTS in 50 mM citrate phosphate buffer at pH 3). Laccase activities were monitored spectrophotometrically in the plate reader by measuring the increase of absorbance of the oxidized products at room temperature.

Optimum pH: reactions of 20 µL enzyme with 180 µL 3 mM ABTS, 3 mM DMP or 9 mM guaiacol solutions (in 100 mM B&R buffer pH 2–10) were carried out by triplicate and the laccase activities monitored in kinetic mode. The relative activities were calculated as a percentage of the maximum activity obtained for each laccase variant.

T_50_ (10 min): 35 µL enzyme aliquots were transferred to 96-well PCR plates, sealed and incubated at a temperature gradient of 30–80 °C during 10 min in a thermocycler (two assays with temperature ramps of 30–55 °C and 55–80 °C were performed). Then, plates were cooled on ice for 10 min and tempered for 5 min. Then, 20 µL samples were transferred to 96-well plates with 180 µL 3 mM ABTS in 50 mM citrate–phosphate buffer pH 3, and laccase activities were measured.

Medium-term temperature stability assay: enzymes were incubated at different times at 60 and 70 °C with 20 mM Tris–HCl buffer at pH 7.5. Then, 20-µL samples taken at different incubation times were added to 96-well plates filled with 180 µL 3 mM ABTS in 50 mM citrate–phosphate buffer pH 3 to measure laccase activity. Laccase half-life values at 60 and 70 °C and thermal inactivation constants were obtained as described [27].

Optimum temperature was determined in the spectrophotometer with a Peltier temperature control using 3 mM ABTS in 50 mM citrate phosphate buffer pH 3 (triplicate samples). The oxidation was followed during the first min of reaction with the substrate pre-incubated at the corresponding temperature.

pH medium-term stability assay: Enzymes were incubated for 1 h at 20 and 30 °C with 20 mM Tris–HCl buffer at pH 10 and 10.5. Then, 20 µL-samples taken at different incubation times were added to 96-well plates filled with 180 µL 3 mM ABTS in 50 mM citrate phosphate buffer pH 3 to measure laccase activity.

Kinetic constants for the oxidation of ABTS (*ε*418 = 36,000 M^−1^ cm^−1^) and DMP (*ε*469 = 27,500 M^−1^ cm^−1^) were measured in triplicate with 20 µL enzyme aliquots added to 230 µL solutions of 3 mM ABTS in 50 mM citrate phosphate pH 3, 3 mM DMP in 100 mM Tris HCl pH 8, 9 or 10 mM. To calculate *K*_m_ and k_*cat*_ values the average Vmax was represented versus substrate concentration and fitted to a single rectangular hyperbola function in SigmaPlot (version 14.0) software, where parameter “a” was equal to k_*cat*_ and parameter “b” was equal to *K*_m_.

### 3D protein modelling

The structure models of the mutated laccases were built with Swiss-Model using 7D5 laccase crystal (PDB entry 6H5Y) as a template, and visually inspected using PyMol Molecular Graphics System (Schrödinger, LLC).

### Determination of redox potential

The published redox potential (E°) for Fe (2,2′-dipyridyl)_2_Cl_3_/Fe(2,2′-dipyridyl)_2_Cl_2_ in 8 mM MES buffer (pH 5.3) was 0.76 V/NHE [36]. The E° (1.11 V vs. NHE) in 12.5 mM Tris–HCl (pH 8) was determined by cyclic voltammetry with a glassy carbon working electrode and an Ag/AgCl/KCl (3 M) reference electrode. The redox potential was referenced to NHE by equation E (vs. NHE) = E (vs. Ag/AgCl/KCl, 3 M) + 0.21.

Laccase redox potential was determined by the poised potential method using the redox couple Fe(2,2′-dipyridyl)_2_Cl_2_/Fe(2,2′-dipyridyl)_2_Cl_3_. Fe(2,2′-dipyridyl)_2_Cl_2_ and Fe(2,2′-dipyridyl)_2_Cl_3_ solutions were prepared freshly, mixing FeCl_2_ (or FeCl_3_) with 2,2′-dipyridyl in the ratio 1:2 in double distilled water. Anaerobicity was achieved by repetitive vacuum-argon cycles, at 4 °C, of solutions, buffers and reaction chamber. All spectrophotometry measurements were carried out under argon atmosphere at 25 °C.

Aliquots of Fe(2,2′-dipyridyl)_2_Cl_3_ solution (0.9–18 µM) were introduced to a solution of Fe(2,2′-dipyridyl)_2_Cl_2_ (9 µM) in MES (8.8 mM, pH 5.5) or Tris–HCl (12.5 mM, pH 8.0) buffer. After introduction of laccase (0.9 µM), oxidation of Fe(2,2′-dipyridyl)_2_Cl_2_ at each titration point was followed by the decrease in absorbance at 522 nm (Fe(2,2′-dipyridyl)_2_Cl_2_: *ε* = 5992 M^−1^ cm^−1^ at pH 5.5 and 5836 M^−1^ cm^−1^ at pH 8.0; Fe(2,2′-dipyridyl)_2_Cl_3_: *ε* = 260 M^−1^ cm^−1^ at pH 5.5 and 225 M^−1^ cm^−1^ at pH 8.0) until equilibrium was reached. The concentration of reduced laccase at equilibrium was considered to be 1/4 of the oxidized Fe(2,2′-dipyridyl)_2_Cl_2_ concentration.

### Kraft lignin oxidation by laccase

Eucalyptus kraft lignin was isolated by the Centre Technique du Papier (Grenoble, France) with LignoBoost from the black liquors of The Navigator Company kraft pulp mill. Kraft lignin (0.5 g/L) was solubilized in 20 mM B&R buffer pH 9–10 and treated with 0.1U/L laccase (100 mL final reaction volume) for 2 and 24 h, at 30 °C and 180 rpm. Same conditions without enzyme served as control lignin. The phenolic and carbonyl contents of laccase-treated and control lignins were spectrophotometrically determined by Folin Ciocalteu Reactive (Abs. 760 nm) and Brady reagent (Abs. 450 nm), respectively [47] *M*_*w*_ distribution of lignin samples were determined by Size Exclusion Chromatography (SEC) using a Superdex75 column pre-equilibrated with 20 mM Britton-Robinson buffer (pH 11.6). Lignin samples were solubilized in NaOH to pH 11.6, centrifuged (13,400 rpm), and injected in the column and Absorbances at 260 and 280 nm were monitored throughout the chromatographic run.

### Heterologous expression in *Komagataella pastoris*

Expression in *Komagataella pastoris* was carried out following Invitrogen^TM^ manual. First, laccase CDS was amplified by PCR with “BstBI long target” and “Ext pJRoC30-R” primers (Additional File 1: Table S3). Then, purification of the PCR product was digested with NotI and BstBI to clone the laccase in the pPICZ-B vector (both, laccase and vector present those restriction sites). Linearized pPICZ-B vector with zeocin resistance was ligated with the digested laccase gene by using T4 DNA ligase, in proportion 1:3 (50 ng of vector and 37.5 ng of laccase) and left overnight at 1 °C.

Ligation product was transformed in *E. coli* and incubated in LB-zeocin plates at 37 °C overnight. Plasmids were isolated with High Pure Plasmid Isolation Kit and digested with SacI in a specific region integrated in AOX (methanol promoter) to linearize the construction and integrate in the genome of *K. pastoris.*

*Komagataella pastoris* X33 strain was transformed following the transformation method provided by the Invitrogen™ manual. Transformed cells were plated in YPD-zeocin and incubated for 2–4 days at 28 °C. The obtained colonies were transferred to BMM ABTS agar plates and incubated for 2–4 days at 2 °C until formation of a green halo (due to oxidation of ABTS) indicated the presence of laccase activity.

After cultivation of a fresh culture in YPD-zeocin incubated for 2–4 days at 28 °C, one colony was transferred into a 50 mL BMMY-zeocin flask and cultivated for 20 h at 28 °C. Glycerol (10 mL) was subsequently added, and thus created glycerol cell stock stored at − 70 °C until further use. The larger scale production fermentation trials were conducted using Infors HT Labfors 5 bioreactors (7.5 l volume), monitored by the bioprocess platform software Eve^®^ (Infors HT). The pH of the fermentation was followed by EasyFerm Plus PHI Arc 425 (Hamilton) via automatic addition of 2 M HCl or 15% ammonium hydroxide. Sufficient oxygenation of the cells was maintained by constant aeration (air) and automatic increase of stirring by two Rushton turbines. Dissolved oxygen (DO) was monitored throughout the cultivation with VisiFerm DO Arc 425 (Hamilton). The main fermentation parameters were kept as follow: temperature 30 °C, dissolved oxygen > 20%, pH 5, agitation: 200–900 rpm, air flow aeration, 0.5–2.5 vvm.

The production process of Li10 laccase was performed through a fed-batch fermentation, consisting of three main phases: the seed train carried over in YPD plate and BMGY flasks, the batch phase initiated in the bioreactor and the fed-batch phase where enzyme production occurs. The seed train was started with the plating of a YPD-zeocin incubated for 48 h at 30 °C and inoculated with the –70 °C cell bank glycerol stock. A pair of isolated colonies were then transferred into BMGY medium flasks and cultivated at 30 °C for 16–24 h. After sufficient growth was noted (OD600 nm > 20), 7% of the fermenter initial fermentation volume was transferred from the BMGY flasks to the fermenter to start the fermentation batch phase. The batch phase was carried over in FBSM media supplemented with PTM1 salts at 30 °C during 18–24 h until exhaustion of the carbon source. This media consists of 40 g/L glycerol, 4.13 g/L potassium hydroxide, 14.9 g/L magnesium sulphate heptahydrate; 18.2 g/L potassium sulphate, 0.93 g/L calcium sulphate, 26.7 mL phosphoric acid (85%) and 4.25 mL/L of PTM1 trace salt (6 g/L copper (II) sulphate pentahydrate, 0.08 g/L sodium iodide, 3 g/L manganese sulfate monohydrate, 0.2 g/L sodium molybdate dihydrate, 0.02 g/L boric acid, 0.5 g/L cobalt chloride, 20 g/L zinc chloride, 65 g/L ferrous sulphate heptahydrate and 5 mL/L sulphuric acid). After exhaustion of the glycerol present in the batch, feed phase was initiated by supplemented glycerol for 4–8 h. Methanol feed was subsequently started and supplemented with copper (6 mM–10 mM) during 80–85 h allowing enzyme production. Downstream process of the fermentation was performed by removal of the cell mass by centrifugation (4000 rpm for 30 min) and freezing of the.

### Laccase-assisted delignification and bleaching of kraft pulps

An oxygen-delignified hardwood kraft pulp (kappa number: 11.5 and brightness: 52% ISO) was provided by Fibre Excellence’s Saint Gaudens mill (France). Li10 laccase crude expressed in *K. pastoris* was applied on this pulp with the following conditions: 100 g of kraft pulp, 14 U of laccase (measured with DMP, pH 8) per g of o.d. pulp, 5 mM methylsyringate, pH 8.5, 65 °C 180 min, 10% pulp consistency. Then the pulp was subjected to the Ep D Ep bleaching sequence. The Ep stage was carried out at 72 °C, 120 min, 10% pulp consistency, 5 kg NaOH/t of o.d. pulp and 10 kg H_2_O_2_/ton of o.d. pulp. At the end, the pulp was washed with tap water on a funnel and then subjected to the D stage in the following conditions: 73 °C, 300 min, 10% consistency and 23 kg ClO_2_/ton of o.d. pulp. After the last Ep stage, the pulp was washed with tap water on a funnel to be further characterized. Pulp kappa number was determined according to standard ISO 302:1981. Pulp brightness was evaluated according to standard ISO 2470:1999 and the residual amounts of ClO_2_ were measured by titration with 0.1 N sodium thiosulfate. The effluents from the different bleaching stages were collected and mixed together. Soluble lignin was determined into the effluent by measurement at 280 nm of an aqueous solution of lignin (10% in water or in 0.25 M NaOH, calibration with vanillin, *ɛ* = 70 g/L cm^−1^).

### Production of medium-density fibreboards (MDF) assisted by laccase

The wood material utilized for board manufacture was maritime pine. The enzyme applied to the wood chips was laccase Li9. The initial protocol based on previous results comprised the de-structuring of chips with a compression screw prior to enzyme application in order to ease the full impregnation of chips by the liquid solution. De-structured chips were submerged into the enzymatic solution during 1 h at 60 °C with 1625 U/kg wood chips of activity (Li9 laccase activity was measured with pH 6 DMP). In a second simplified protocol the de-structuration step was removed and the immersion of chips into the enzymatic solution was applied for 2 h at same temperature. De-structured chips were steamed for 10 min, whereas non-de-structured ones were steamed for 20 min in order to soften lignin and facilitate fibre separation. Controls were carried out at the same conditions without enzyme addition. Besides, control chips with no water immersion (with and without compression screw) were also processed for MDF production.

The chips were introduced in the refiner for defibering under 6 bar pressure with feeding adapted for flow to be compatible with the equipment. Fibres were produced in a pressurized 12’ Andritz refiner equipped with Durametal plates (Ref. 12SA001) based on the equipment used by the industry for manufacturing MDF fibres. Fibre pressing was performed on a heating hydraulic press with a Plate surface of 60 × 60 cm^2^, under 200 °C. Wood panels were characterized on equipment delivered by Instron. The fibres were evacuated through the blow line and recovered after separation cyclone. Power consumption was acquired during processing and energy was calculated based on the corresponding flow. Industrial production of MDF comprises several steps that were reproduced in the laboratory (Additional File 1: Fig. S8). Resin was introduced at 12% in mass. Two ranges of densities were aimed at in order to interpolate results. Density of the boards was measured according to EN323, internal bond according to EN319 and water swelling according to EN317 standards.

## Conclusions

For the first time, fungal laccases of high redox potential with alkaliphilic and thermophilic properties have been tailor-made to supply more sustainable and cleaner means of production in wood conversion processes. The enzymes designed through directed evolution and rational design are able to oxidize lignin at pH 10 (something never reported before for this type of enzymes), and show optimal activities at 70 °C and notably improved thermal-resistance. The application of the tailor-made extremophilic laccases in kraft pulp bleaching and fibreboard manufacture saves energy and chemicals, while increases process efficiency. These results are the subject of a patent application (EP21382978, PCT/EP2022/080219).

### Supplementary Information

**Additional file 1.** **Table S1.** Mutations selected during the directed evolution of 7A12 (green) and RY2 (blue) laccases towards alkaliphilicity, the accumulation of which confers better phenol oxidation at increasing pH values to the laccase variants successively obtained in the two lineages; **Table S2.** Amino acid residues in which parent laccases 7A12 and RY2 differ, and amino acid substitutions accumulated in their selected evolved variants (in green for 7A12 lineage and in blue for RY2 lineage). Laccase conserved motifs are highlighted in orange; **Table S3.** Primers used for SM and CSM on target amino acid residues of laccase; **Figure S1.** Thermal tolerance of the engineered laccases determined as T_50_ (10 min) curves for 7A12 lineage (A) and RY2 lineage (B); **Figure S2.** Sequence logo for residues near T1 Cu from the multiple alignment of sensu-stricto laccases of 52 genomes of basidiomycete fungi; **Figure S3.** Close-up of the 3D-structure model of 7A12 laccase showing the catalytic site with the four copper ions as blue spheres and the residues coordinating the coppers as white sticks. Based on PDB entry 6H5Y; **Figure S4.** Improvement of activity with DMP at pH 8 in variant Li11 (D263V), and in mutants D263G and D263A, obtained by SM mutagenesis of D263 in Li10 laccase. Activity of Li10 is indicated as a dotted line; **Figure S5.** Shift of optimal pH for the oxidation of guaiacol in the alkaliphilic variants of RY2 lineage; parent RY2 (black circles), Li10 (white circles), Li11 (black inverted triangles), C-LeB (white triangles) and Mol3 (black squares) laccases; **Figure S6.** Stability at pH 10 (A) and 10.5 (B) of Li10 (white circles), Li11 (black inverted triangles) and C-LeB (white triangles) laccases (crude enzymes). Each point represents the average of three independent experiments ± standard deviation. **Figure S7.** Flask production by *S. cerevisiae* of Mol3 laccase with its signal peptide, α9H2 leader [27], that has been used so far with all laccase variants in this study (black triangles) or with the optimised signal peptide αOPT (white triangles) developed in a previous work [29]. Laccase activity (U/L) was measured with ABTS pH 3. Error bars indicate standard derivation of three flask replicates; **Figure S8.** Steps for chip preparation and production of MDF.

## Data Availability

Not applicable.
