# Supplementary material for: Tailor-made alkaliphilic and thermostable fungal laccases for industrial wood processing
Source: Biotechnol Biofuels Bioprod. 2022 Dec 29;15:149. doi: 10.1186/s13068-022-02247-2 (PMC9798632; doi:10.1186/s13068-022-02247-2)
Supplement: Supplementary file 1 — Additional file 1. Table S1. Mutations selected during the directed evolution of 7A12 (green) and RY2 (blue) laccases towards alkaliphilicity, the accumulation of which confers better phenol oxidation at increasing pH values to the laccase variants successively obtained in the two lineages; Table S2. Amino acid residues in which parent laccases 7A12 and RY2 differ, and amino acid substitutions accumulated in their selected evolved variants (in green for 7A12 lineage and in blue for RY2 lineage). Laccase conserved motifs are highlighted in orange; Table S3. Primers used for SM and CSM on target amino acid residues of laccase; Figure S1. Thermal tolerance of the engineered laccases determined as T50 (10 min) curves for 7A12 lineage (A) and RY2 lineage (B); Figure S2. Sequence logo for residues near T1 Cu from the multiple alignment of sensu-stricto laccases of 52 genomes of basidiomycete fungi; Figure S3. Close-up of the 3D-structure model of 7A12 laccase showing the catalytic site with the four copper ions as blue spheres and the residues coordinating the coppers as white sticks. Based on PDB entry 6H5Y; Figure S4. Improvement of activity with DMP at pH 8 in variant Li11 (D263V), and in mutants D263G and D263A, obtained by SM mutagenesis of D263 in Li10 laccase. Activity of Li10 is indicated as a dotted line; Figure S5. Shift of optimal pH for the oxidation of guaiacol in the alkaliphilic variants of RY2 lineage; parent RY2 (black circles), Li10 (white circles), Li11 (black inverted triangles), C-LeB (white triangles) and Mol3 (black squares) laccases; Figure S6. Stability at pH 10 (A) and 10.5 (B) of Li10 (white circles), Li11 (black inverted triangles) and C-LeB (white triangles) laccases (crude enzymes). Each point represents the average of three independent experiments ± standard deviation. Figure S7. Flask production by S. cerevisiae of Mol3 laccase with its signal peptide, α9H2 leader [27], that has been used so far with all laccase variants in this study (blac [file 13068_2022_2247_MOESM1_ESM.docx]

**Supplementary information**

Tailor-made extremophilic fungal laccases for industrial wood processing

David Rodríguez-Escribano, Rocío Pliego-Magán, Felipe de Salas, Pablo Aza, Patrizia Gentili, Petri Ihalainen, Thomas Levée, Valérie Meyer, Michel Petit-Conil, Sandra Tapin-Lingua Michael Lecourt, and Susana Camarero

**Results**

**Table S1**. Mutations selected during the directed evolution of 7A12 (green) and RY2 (blue) laccases towards alkaliphilicity, the accumulation of which confers better phenol oxidation at increasing pH values to the laccase variants successively obtained in the two lineages.

|  | SM 263 | SM F392 | ep PCR/DM | SM 454 | CSM 456-457-458 | | | SM F460 |
| --- | --- | --- | --- | --- | --- | --- | --- | --- |
| Variant | **263** | **392** | **396** | **454** | **456** | **457** | **458** | **460** |
| **7A12** | **N** | **F** | **F** | **E** | **L** | **E** | **A** | **F** |
| **RK6** | **N** | **F** | **I** | **E** | **L** | **E** | **A** | **F** |
| **RK7** | **N** | **F** | **I** | **P** | **L** | **E** | **A** | **F** |
| **RK8** | **N** | **N** | **I** | **P** | **L** | **E** | **A** | **F** |
| 1 | N | Y | I | P | L | E | A | F |
| 2 | N | T | I | P | L | E | A | F |
| 3 | N | S | I | P | L | E | A | F |
| **RK9** | **N** | **N** | **I** | **P** | **I** | **D** | **L** | **F** |
| 1 | N | N | I | P | M | D | T | F |
| **RY2** | **D** | **F** | **F** | **P** | **L** | **D** | **A** | **F** |
| **Li9** | **D** | **N** | **I** | **P** | **L** | **D** | **A** | **F** |
| 1 | D | Y | I | P | L | D | A | F |
| 2 | D | T | I | P | L | D | A | F |
| **Li10** | **D** | **N** | **I** | **P** | **L** | **D** | **A** | **M** |
| 1 | D | N | I | P | L | D | A | L |
| **Li11** | **V** | **N** | **I** | **P** | **L** | **D** | **A** | **M** |
| 1 | G | N | I | P | L | D | A | M |
| 2 | A | N | I | P | L | D | A | M |
| **C-LeB** | **V** | **N** | **I** | **P** | **L** | **D** | **L** | **M** |
| 1 | V | N | I | P | L | E | A | M |
| 2 | V | N | I | P | M | D | R | M |
| 3 | V | N | I | P | M | E | A | M |
| 4 | V | N | I | P | M | D | A | M |
| 5 | V | N | I | P | L | D | V | M |
| 6 | V | N | I | P | L | E | L | M |
| 7 | V | N | I | P | M | D | I | M |
| 8 | V | N | I | P | M | D | A | M |

**Table S2**. Amino acid residues in which parent laccases 7A12 and RY2 differ, and amino acid substitutions accumulated in their selected evolved variants (in green for 7A12 lineage and in blue for RY2 lineage). Laccase conserved motifs are highlighted in orange.

**Table S3**. Primers used for SM and CSM on target amino acid residues of laccase.

| **Primer** | **Sequence** |
| --- | --- |
| Ext pJRoC30-F | CTGGGGTAATTAATCAGCGAAGC |
| Ext pJRoC30-R | CCAAAACCTTCTCAAGCAAGG |
| 454SM Fw | CACATTGACNNKCACCTTGACG |
| 454SM Rv | CGTCAAGGTGMNNGTCAATGTG |
| 392SM-Fw | CCC GGC NNK CCG CAC CCC ATC CAC TTG |
| 392SM-Rw | CAA GTG GAT GGG GTG CGG MNN GCC GGG |
| 460 Li9-Fw | GGC NNK GCT GTA GTC ATG GCC |
| 460 Li9-Rw | GGC CAT GAC TAC AGC MNN GCC |
| LEA Fw | AT TAC CCT CAC NTS VMS VNA AGG CAT GGC T |
| LEA Rw | AGC CAT GCC TNB SKB SAN GTG AGG GTC AAT |
| N263DFW | CCTTCCCGACTCCGGGACCA |
| N263DRV | TGGTCCCGGAGTCGGGAAGG |
| V413 FW | GGG AGC AGC GTC TAC AAC TAC GCG |
| V413 RV | CGC GTA GTT GTA GAC GCT GCT CCC |
| S291T FW | ACC ACG ACC CAG ACG CCG TCG |
| S291T RV | CGA CGG CGT CTG GGT CGT GGT |
| D457E FW | ACC TTG AGG CTG GGT T |
| D457E RV | AAC CCA GCC TCA AGG T |
| A461T FW | TTC ACG GTC GTC ATG |
| A461T RV | CAT GAC GAC CGT GAA |
| T468I FW | GAG GAC ATT CCC GAC |
| T468I RV | GTC GGG AAT GTC CTC |
| TopDown424Fw | TAC CGC GAC GTC GTC TCT ACG GGC |
| TopDown424Rv | GCC CGT AGA GAC GAC GTC GCG GTA |
| TopDown426Fw | TAC CGC GAC ACT GTC AAC ACG GGC |
| TopDown426Rv | GCC CGT GTT GAC AGT GTC GCG GTA |
| TopDown429Fw | ATC CGG TTC ACT ACG GAC AAC CCA GGC CCG |
| TopDown429Rv | CGG GCC TGG GTT GTC CGT AGT GAA CCG GAT |
| TopDown430Fw | ACG GGC TCG GAT GGG GAC AAC GTC |
| TopDown430Rv | GAC GTT GTC CCC ATC CGA GCC CGT |
| TopDown439Fw | ACG GGC ACT CCC GGG GAC AAC GTC |
| TopDown439Rv | GAC GTT GTC CCC GGG AGT GCC CGT |
| TopDown441Fw | ACG ATC CGG TTC AGG ACG AAT AAC CCA GGC CCG |
| TopDown441Rv | CGG GCC TGG GTT ATT CGT CCT GAA CCG GAT CGT |
| TopDown441Fw | ACG ATC CGG TTC AGG ACG AAT AAC CCA GGC CCG |
| TopDown441Rv | CGG GCC TGG GTT ATT CGT CCT GAA CCG GAT CGT |
| BottomUp V424T Fw | TAC CGC GAC ACT GTC AAC ACG GGC |
| BottomUp V424T Rv | GCC CGT GTT GAC AGT GTC GCG GTA |
| BottomUp S429T Fw | AAC ACG GGC ACT CCC GGG GAC AAC |
| BottomUp S429T Rv | GTT GTC CCC GGG AGT GCC CGT GTT |
| BottomUp D441N Fw | AGG ACG AAT AAC CCA GGC CCG TGG |
| BottomUp D441N Rv | CCA CGG GCC TGG GTT ATT CGT CCT |
| BottomUp V424T BottomUp S429T Fw | CGC GAC ACT GTC AAC ACG GGC ACT CCC GGG |
| BottomUp V424T BottomUp S429T Rv | CCC GGG AGT GCC CGT GTT GAC AGT GTC GCG |
| BottomUp V424T BottomUp D441N Fw | GAC ACT GTC AAC ACG GGC TCG CCC GGG GAC AAC GTC ACG ATC CGG TTC AGG ACG AAT AAC |
| BottomUp V424T BottomUp D441N Rv | GTT ATT CGT CCT GAA CCG GAT CGT GAC GTT GTC CCC GGG CGA GCC CGT GTT GAC AGT GTC |
| BottomUp S429T BottomUp D441N Fw | ACG GGC ACT CCC GGG GAC AAC GTC ACG ATC CGG TTC AGG ACG AAT AAC |
| BottomUp S429T BottomUp D441N Rv | GTT ATT CGT CCT GAA CCG GAT CGT GAC GTT GTC CCC GGG AGT GCC CGT |
| BottomUp Mol3 Fw | GAC ACT GTC AAC ACG GGC ACT CCC GGG GAC AAC GTC ACG ATC CGG TTC AGG ACG AAT AAC |
| BottomUp Mol3 Rv | GTT ATT CGT CCT GAA CCG GAT CGT GAC GTT GTC CCC GGG AGT GCC CGT GTT GAC AGT GTC |


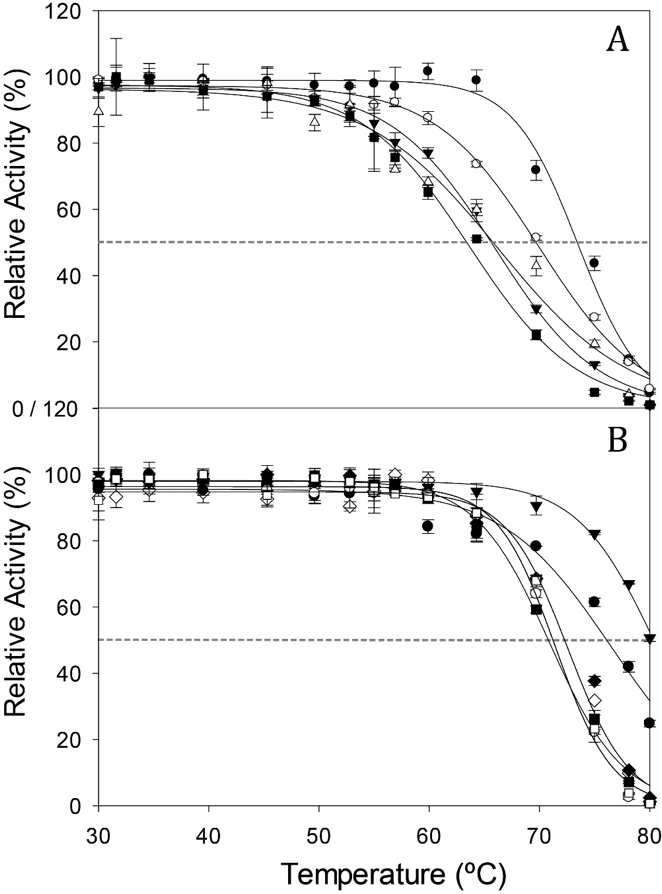


**Figure S1.** Thermal tolerance of the engineered laccases determined as T_50_ (10 min) curves for 7A12 lineage (A) and RY2 lineage (B).7A12 lineage: parent 7A12, (black circles), RK6 (white circles), RK7 (white triangles), RK8 (black inverted triangles) and RK9 (black squares). RY2 lineage: parent RY2 (white diamonds), Li9 (black diamonds), Li10 (white circles), Li11 (black squares), C-LeB (white squares), Mol2 (black circles) and Mol3 (black inverted triangles).


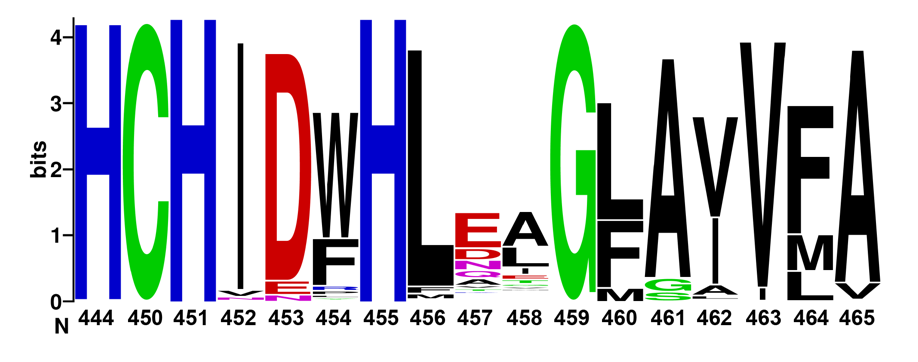


**Figure S2.** Sequence logo for residues near T1 Cu from the multiple alignment of sensu-stricto laccases of 52 genomes of basidiomycete fungi.


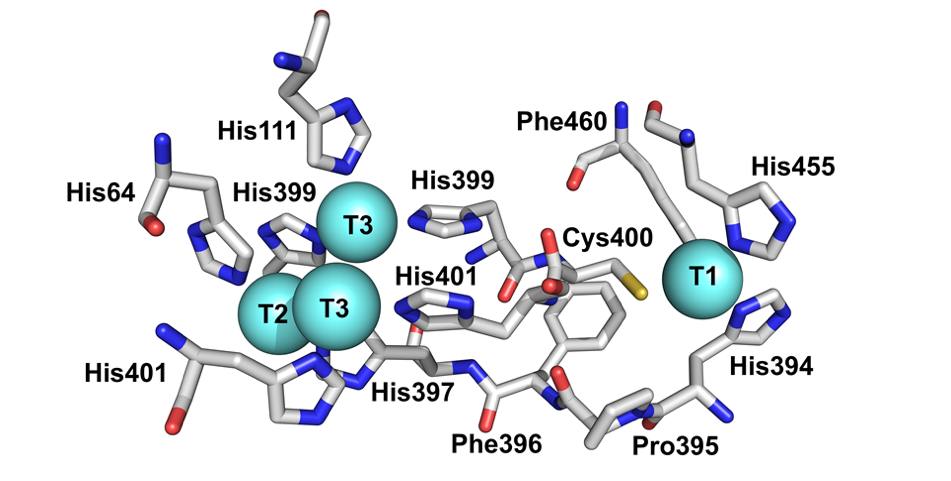


**Figure S3.** Close-up of the 3D-structure model of 7A12 laccase showing the catalytic site with the four copper ions as blue spheres and the residues coordinating the coppers as white sticks. Based on PDB entry 6H5Y.


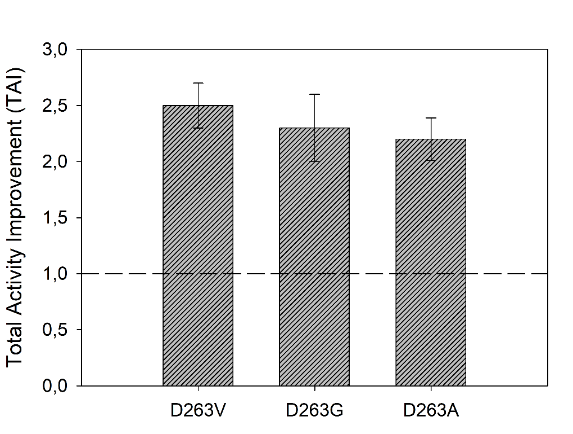


**Figure S4.** Improvement of activity with DMP at pH 8 in variant Li11 (D263V), and in mutants D263G and D263A, obtained by SM mutagenesis of D263 in Li10 laccase. Activity of Li10 is indicated as a dotted line.


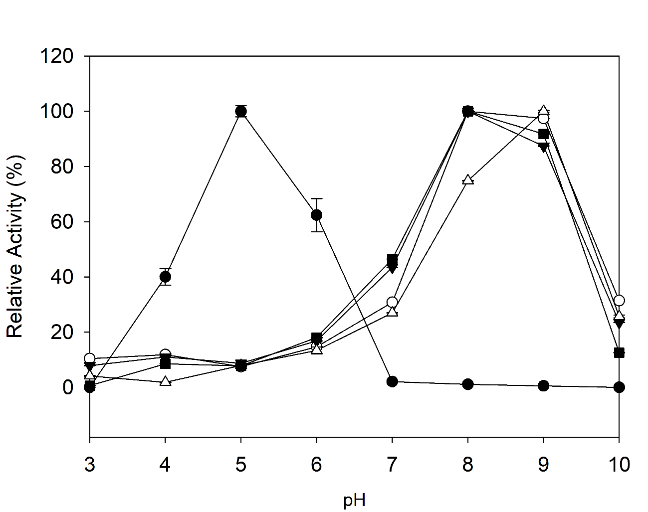


**Figure S5**. Shift of optimal pH for the oxidation of guaiacol in the alkaliphilic variants of RY2 lineage; parent RY2 (black circles), Li10 (white circles), Li11 (black inverted triangles), C-LeB (white triangles) and Mol3 (black squares) laccases.


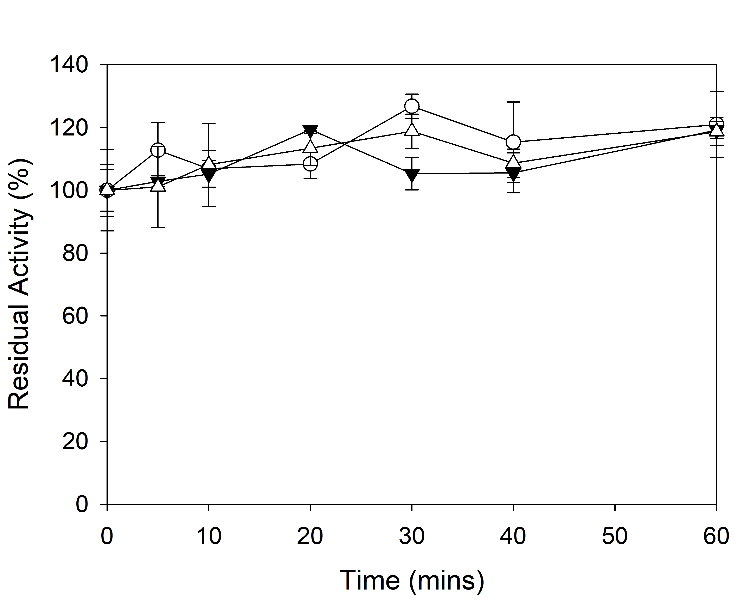

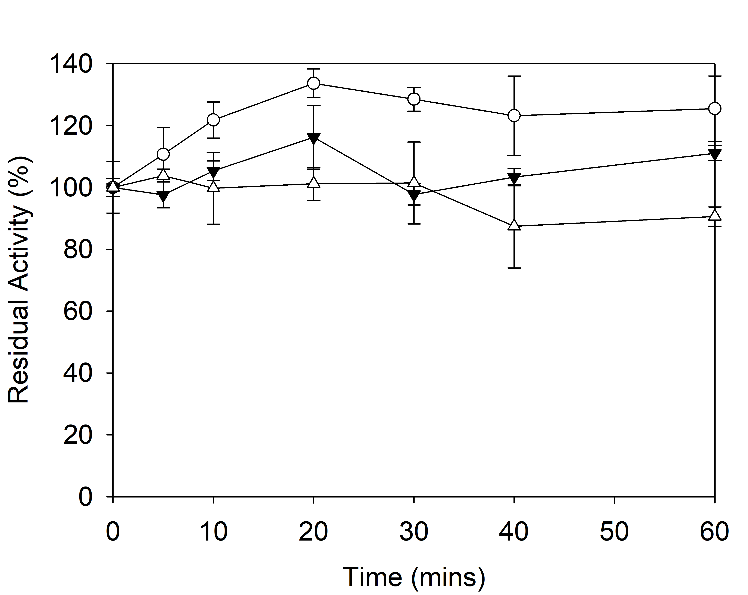


**
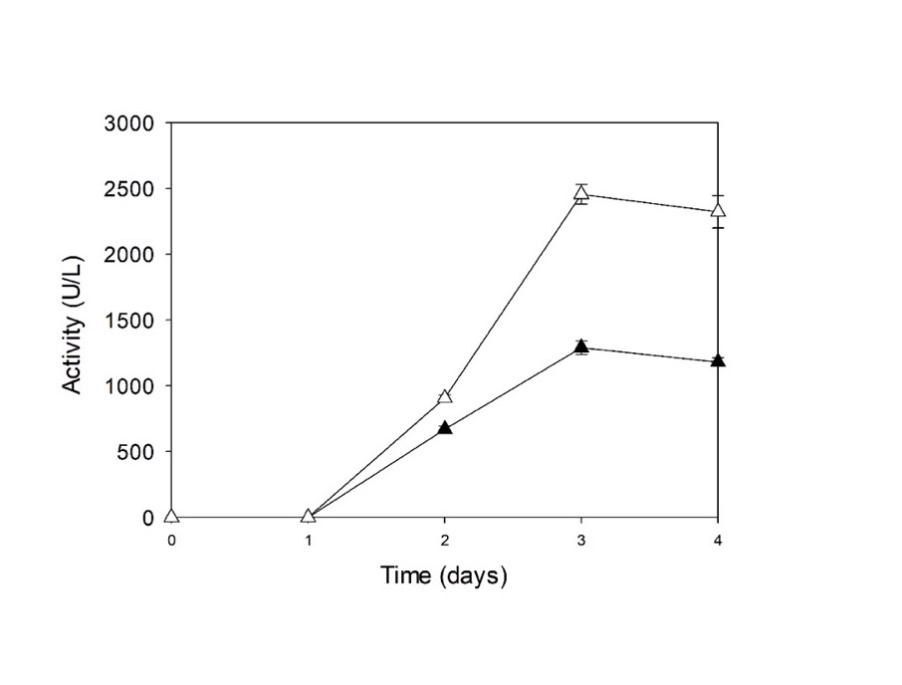
Figure S6** Stability at pH 10 (A) and 10.5 (B) of Li10 (white circles), Li11 (black inverted triangles) and C-LeB (white triangles) laccases (crude enzymes). Each point represents the average of three independent experiments ± standard deviation.

**Figure S7**. Flask production by *S. cerevisiae* of Mol3 laccase with its signal peptide, α_9H2_ leader [27], that has been used so far with all laccase variants in this study (black triangles) or with the optimised signal peptide α_OPT_ (white triangles) developed in a previous work [29]. Laccase activity (U/L) was measured with ABTS pH 3. Error bars indicate standard derivation of three flask replicates.

| 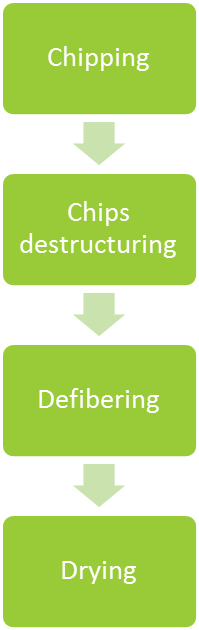 | 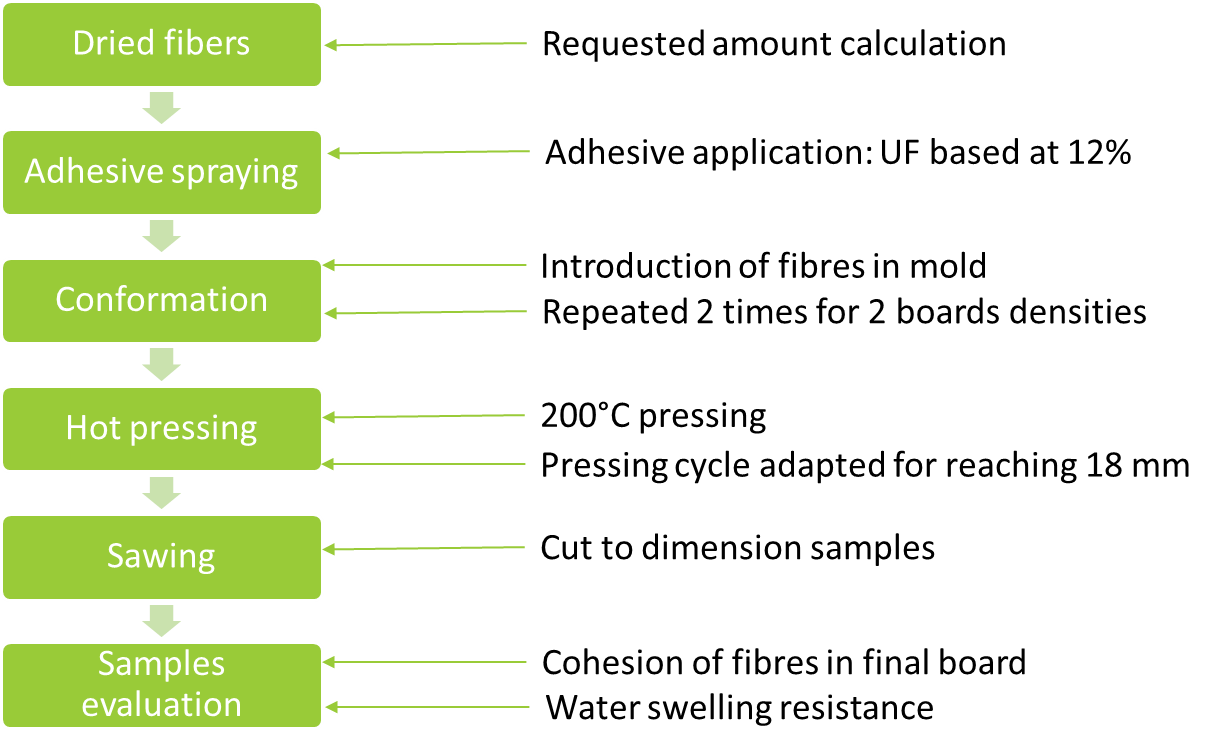 |
| --- | --- |
|  |  |

**Figure S8.** Steps for chip preparation and production of MDF.
